# Global warming makes nitrogen oxide abatement key to ozone pollution mitigation

**DOI:** 10.1126/sciadv.aea4124

**Published:** 2026-07-17

**Authors:** Wenjie Wang, Hang Su, David D. Parrish, Ruijing Ni, Xin Li, Fengxia Bao, Hongli Wang, Cheng Huang, Jonathan Williams, Hong Liao, Keding Lu, Yuanhang Zhang, Yafang Cheng

**Affiliations:** ^1^ Max Planck Institute for Chemistry, Mainz 55128, Germany.; ^2^ Nanjing-Helsinki Institute in Atmospheric and Earth System Sciences, Nanjing University, Suzhou 215163, China.; ^3^ State Key Laboratory of Atmospheric Environment and Extreme Meteorology, Institute of Atmospheric Physics, Chinese Academy of Sciences, Beijing 100029, China.; ^4^ David D. Parrish, LLC, Boulder, CO 80303, USA.; ^5^ State Key Joint Laboratory of Environmental Simulation and Pollution Control, College of Environmental Sciences and Engineering, Peking University, Beijing 100871, China.; ^6^ State Environmental Protection Key Laboratory of Formation and Prevention of Urban Air Pollution Complex, Shanghai Academy of Environmental Sciences, Shanghai 200233, China.; ^7^ Climate and Atmosphere Research Center, The Cyprus Institute, Nicosia, Cyprus.; ^8^ Jiangsu Key Laboratory of Atmospheric Environment Monitoring and Pollution Control, Jiangsu Collaborative Innovation Center of Atmospheric Environment and Equipment Technology, School of Environmental Science and Engineering, Nanjing University of Information Science and Technology, Nanjing 210044, China.

## Abstract

Global warming has become a critical factor affecting surface ozone (O_3_) pollution. The effectiveness of emission reductions for O_3_ pollution mitigation largely relies on the sensitivity of O_3_ production to its precursors. However, how global warming affects the O_3_ sensitivity remains unclear, posing a grand challenge in developing effective strategies for O_3_ pollution mitigation. Here, by using comprehensive ambient observations to constrain model simulations, we show a strong temperature dependence of O_3_ sensitivity over a large temperature range (−5° to 37°C) in Chinese cities. O_3_ sensitivity shifts toward the nitrogen oxide–limited regime at higher temperatures. Temperature change alters the reaction regime by primarily modulating the rate coefficient of radical termination, rather than that of radical cycling, and by altering emissions of biogenic volatile organic compounds. Overall, future global warming will accelerate the transition of O_3_ sensitivity toward the nitrogen oxide–limited regime, amplifying the benefits of nitrogen oxide emission abatement in mitigating O_3_ pollution in urban areas worldwide.

## Teaser

Global warming amplifies the benefits of nitrogen oxide emission abatement in mitigating O_3_ pollution.

## INTRODUCTION

Near-ground ozone (O_3_) is a major air pollutant that is harmful to human health, vegetation, and ecosystem productivity (*1*–*3*). Global warming affects the photochemical production and ambient concentrations of O_3_ by altering biogenic emissions, chemical reaction rates, water vapor (thereby HO*_X_* chemistry), and physical dilution in the atmosphere (*4*, *5*). Under the warming climate, more frequent occurrence of extremely high temperatures exacerbates O_3_ pollution (*6*).

Volatile organic compounds (VOCs) and nitrogen oxides (NO*_X_* ≡ NO + NO_2_) are important precursors of O_3_ pollution (*3*). The O_3_ sensitivity regime, i.e., whether O_3_ production is NO*_X_* limited or VOC limited (i.e., NO*_X_* saturated), largely regulates the effectiveness of emission reductions for O_3_ mitigation (*7*–*9*). In the NO*_X_*-limited regime, controlling NO*_X_* is more effective in reducing O_3_ than controlling VOCs, whereas, in the VOC-limited regime, controlling VOCs is more effective than controlling NO*_X_*. Regions worldwide affected by O_3_ pollution have implemented ongoing reductions in O_3_ precursor emissions, resulting in notable changes in O_3_ sensitivity across many cities. As a result, for many cities in North America and Europe, summertime O_3_ sensitivity has changed from a VOC-limited regime to a NO*_X_*-limited regime over the past three decades (*10*, *11*). In contrast, many cities in developing countries such as China and India are still in a VOC-limited regime (*8*, *12*, *13*). Previous studies have shown that heat-wave events increased O_3_ production by boosting anthropogenic and biogenic emissions (*4*, *14*–*18*), whereas the temperature-driven change of the O_3_ sensitivity regime and its impact on the effectiveness of emission reductions remain elusive, posing a grand challenge in designing effective strategies for O_3_ pollution mitigation.

Here, by applying ambient observations combined with an observation-based box model and a chemical transport model (GEOS-Chem), we comprehensively investigate the impact of temperature on O_3_ sensitivity in real atmospheric environments and then explore its implications for O_3_ pollution mitigation under warming climate. A critically important aspect of this study is the availability of measurements of VOCs and major oxidants including hydroxyl radicals (OH) and peroxyl radicals (HO_2_ and RO_2_), which provide strong constraints on the model simulation results.

## RESULTS

### The temperature dependence of O_3_ sensitivity between seasons

To depict a clear framework of the relationship between temperature and O_3_ sensitivity over a large temperature range, we investigate the difference in O_3_ sensitivity between a wintertime month (February, on average of 3°C) and a summertime month (July, on average of 28.5°C) in 2019 at the Peking University Urban Atmospheric Environment Monitoring Station (PKUERS) site in Beijing. The comparison between the two time periods accounts for seasonal differences in temperature, water vapor, solar radiation, and concentrations of O_3_ precursors. Beijing is one of China’s megacities and suffers from severe O_3_ pollution. Its experience in the control of O_3_ pollution can serve as a useful reference for other Chinese cities that are still experiencing rapidly increasing O_3_ concentrations. The PKUERS site is a representative urban site in Beijing, where the concentrations of O_3_ and its precursors are comparable to citywide averages (*12*).

**Fig. 1.**
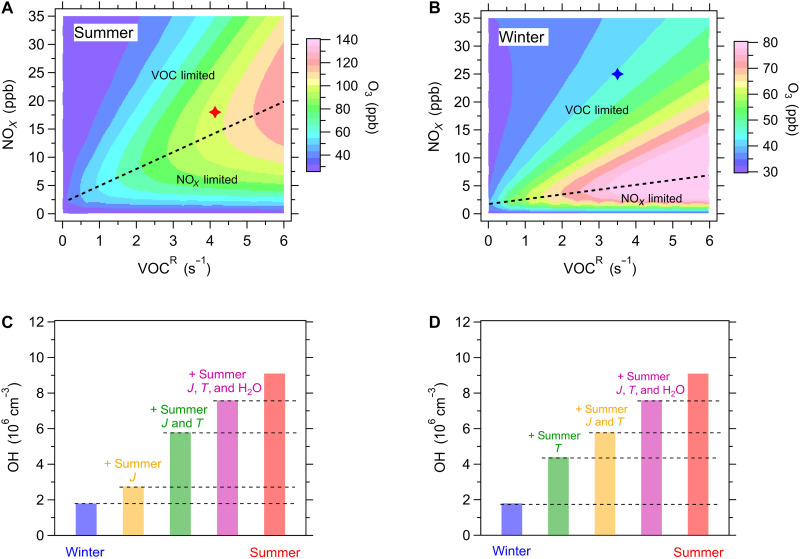
The contrast of O_3_ sensitivity between summer and winter in Beijing. (**A** and **B**) Box model–simulated isopleth diagrams for O_3_ as a function of NO*_X_* and VOCs in summer (A) and winter (B), respectively. VOCs are quantified by OH reactivity of VOCs (VOC^R^) (see the “Measurement data” section). The averages of NO*_X_* and VOC^R^ in summer (red symbol) and winter (blue symbol) are marked on the isopleth diagrams. The dashed lines indicate the ridge lines that distinguish between VOC-limited and NO*_X_*-limited regimes. (**C** and **D**) Box model–simulated peak OH concentrations. In (C), five scenarios are simulated: winter (blue bar); summer (red bar); winter with summertime photolysis frequencies (*J*) (orange bar); winter with summertime photolysis frequencies and temperature (*T*) (green bar); and winter with summertime photolysis frequencies, temperature, and water vapor (purple bar). In (D), five scenarios are simulated: winter (blue bar); summer (red bar); winter with summertime temperature (green bar); winter with summertime photolysis frequencies and temperature (orange bar); and winter with summertime photolysis frequencies, temperature, and water vapor (purple bar). For (C) and (D), the impact of temperature on OH includes temperature-induced changes in reaction rate constants and isoprene concentrations.

We investigate the O_3_ sensitivity change by applying a box model with the detailed Master Chemical Mechanism (MCM) v3.3.1 chemistry scheme (*19*) (see the “Observation-based photochemical box model” section). The inputs of the box model include comprehensive observations of C_2_-C_10_ nonmethane hydrocarbons (NMHCs), inorganic gases (NO*_X_*, carbon monoxide, and sulfur dioxide), aerosol surface-area concentrations, and meteorological factors, such as temperature, relative humidity, air pressure, and photolysis frequencies. The model simulation accounts for the diurnal evolution of the planetary boundary layer, the exchange of O_3_ between near surface air and the residual layer and the heterogeneous uptake of HO_2_ radicals and reactive nitrogen species onto aerosols (*12*). Previous studies have demonstrated its good performance in the diagnosis of O_3_ sensitivity given that O_3_ sensitivity primarily depends on local photochemistry (*12*, *20*). In addition, the model well simulates key photochemical products and RO*_X_* radicals with biases of less than 25% (figs. S1 and S2). Figure 1 (A and B) shows the simulated isopleth diagrams for O_3_ as a function of NO*_X_* and OH reactivity of VOCs (VOC^R^) in summer and winter. Although O_3_ sensitivity falls into a VOC-limited regime in both winter and summer, the O_3_ sensitivity in summer is closer to the transitional region between the two regimes (dashed lines in Fig. 1, A and B) than in winter. Shanghai, another megacity in southern China, exhibits a similar seasonal contrast in O_3_ sensitivity to Beijing (fig. S3). The contrasting O_3_ sensitivity between summer and winter has been supported by satellite measurements of the formaldehyde to NO_2_ ratio, a known metric of O_3_ sensitivity (*13*), and by regional air-quality model simulations for Chinese urban areas (*21*).

Essentially, O_3_ sensitivity depends on the dominant loss pathways of RO*_X_* (RO*_X_* ≡ OH + HO_2_ + RO_2_) radicals. O_3_ production is NO*_X_* limited if the self-reaction of peroxyl radicals dominates the RO*_X_* sink and VOC limited if the reaction of NO_2_ with OH dominates (*8*, *22*). Accordingly, the ratio of OH + NO_2_ reaction rate to the total rate of the two RO*_X_* sinks, defined as φ, is used to identify O_3_ sensitivity regimes, as shown in Eq. 1 (text S1). O_3_ production is NO*_X_* limited if φ is lower than 0.5; otherwise, it is VOC limited. A lower φ value indicates a higher degree of NO*_X_*-limited regime

$$\varphi =\frac{k_{\text{OH}+{\text{NO}}_{2}}\left[\text{OH}\right]\left[{\text{NO}}_{2}\right]}{k_{{\text{HO}}_{2}+{\text{RO}}_{2}}\left[{\text{HO}}_{2}\right]\left[{\text{RO}}_{2}\right]+k_{{\text{HO}}_{2}+{\text{HO}}_{2}}\left[{\text{HO}}_{2}\right]\left[{\text{HO}}_{2}\right]+k_{\text{OH}+{\text{NO}}_{2}}\left[\text{OH}\right]\left[{\text{NO}}_{2}\right]}$$ (1)

where $k_{\text{OH}+{\text{NO}}_{2}}$, $k_{{\text{HO}}_{2}+{\text{RO}}_{2}}$, and $k_{{\text{HO}}_{2}+{\text{HO}}_{2}}$ are reaction rate constants of reactions OH + NO_2_, HO_2_ + RO_2_, and HO_2_ + HO_2_, respectively. We calculate φ using the box model simulation constrained by comprehensive observations in winter and summer in Beijing. For this calculation, OH, HO_2_, and RO_2_ are simulated by the box model. The daytime (08:00 to 18:00) average of φ is used in this study as photochemical O_3_ production primarily occurs during daylight hours. The uncertainty in the simulated φ arising from uncertainties in key reaction rate constants associated with radical termination and cycling is estimated to be 12% (see the “The uncertainty of the model simulation” section).

Given that the OH radical is a key oxidant that determines O_3_ sensitivity (*12*, *23*, *24*), we investigate the impact of temperature on OH concentrations with the box model by accounting for the changes in reaction rate constants and isoprene emissions induced by temperature change. As shown in Fig. 1 (C and D), the change in temperature has a larger effect on OH concentrations than the changes in photolysis frequencies and humidity, with the rise in temperature from winter to summer increasing OH. Note that the only difference between panels (C) and (D) of Fig. 1 is the order in which changes of temperature and photolysis frequency are applied, confirming that temperature change plays a key role regardless of the order. The combined effects of changes in temperature, humidity, and photolysis frequencies cannot fully explain the observed difference in OH concentrations between winter and summer, as indicated by the difference between the purple and red bars in Fig. 1 (C and D). This residual difference is attributed to additional influences from changes in concentrations of VOCs, NO*_X_*, and oxidation products.

**Fig. 2.**
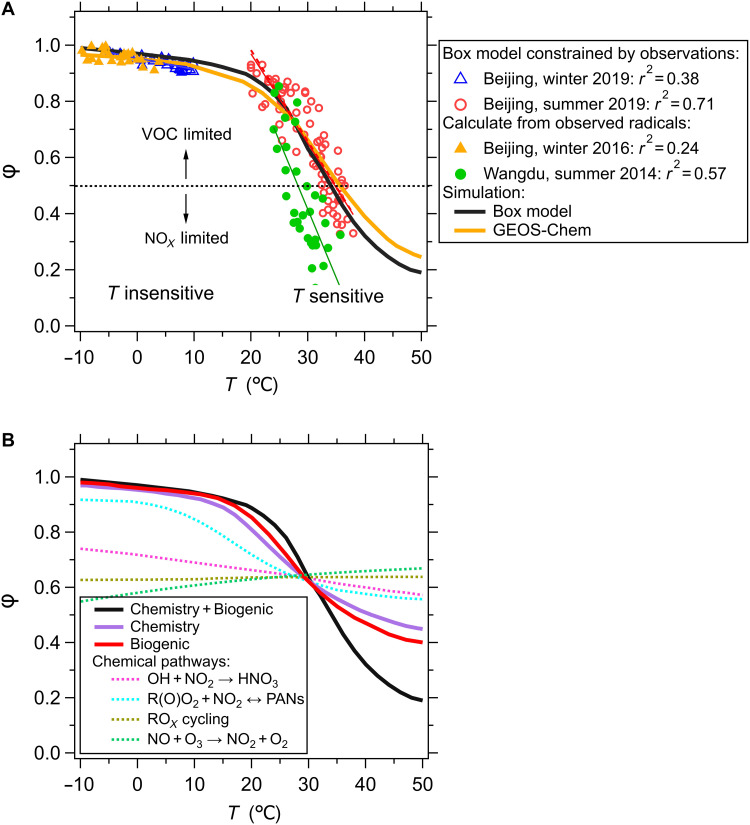
The mechanism driving the impact of temperature on O_3_ sensitivity. (**A**) Correlation between φ and temperature. φ Values in winter (blue triangles) and summer (red circles) in Beijing are calculated by the box model with constraints of observations of O_3_, O_3_ precursors, and meteorological factors in Beijing. As a comparison, φ values in winter in Beijing (orange solid triangles) and in summer in Wangdu (green solid circles) calculated from observed NO_2_, OH, HO_2_, and RO_2_ radicals are presented. Each data point represents a daily daytime average. Also presented is the temperature dependence of φ in Beijing simulated by the box model (black line) and the GEOS-Chem model (orange line). *r*^2^, coefficient of determination. (**B**) The temperature dependence of φ induced by chemistry and biogenic VOC emissions (black line), chemistry alone (purple line), and biogenic VOC emissions alone (red line), and individual chemical reactions (dashed lines), which is simulated by the box model.

As shown in Fig. 2A, the box model–simulated φ shows a moderate negative correlation with temperatures in winter (blue triangles) and a notable negative correlation with temperatures in summer (red circles), consistently decreasing as temperatures increase from −10° to 37°C. As a comparison, we also calculate φ values by using observed concentrations of NO_2_, OH, HO_2_, and RO_2_ in winter 2016 in Beijing (orange solid triangles) and in summer 2014 in Wangdu (green solid circles), a city that is 170 km away from Beijing, showing a similar temperature dependence to the results of the box model simulations.

### The mechanism driving the temperature dependence of O_3_ sensitivity

Although previous studies have shown that rising temperature can increase O_3_ production (*4*, *14*–*18*, *25*), how global warming affects O_3_ sensitivity regime is still not well understood. Here, we explore the underlying chemical mechanism causing the temperature dependence of O_3_ sensitivity. A change in temperature can alter the reaction rate constants of many reactions involved in cycling and termination of RO*_X_* radicals (table S3) (*26*). Nevertheless, it remains unclear how the net effect of these changes affects O_3_ sensitivity. In the model simulation, we take the summer scenario as a base scenario and conduct sensitivity tests by changing the reaction rate constant of the investigated reaction over a large temperature range (−10° to 50°C) (see the “Evaluating the mechanism driving the temperature dependence of φ” section). As shown in Fig. 2B and fig. S4, temperature affects O_3_ sensitivity mainly through changing the reaction rate constants of radical termination, rather than changing the reaction rate constants of radical cycling, although radical cycling is considered the most important process for O_3_ production (*12*, *23*). Specifically, higher temperatures accelerate the thermal decomposition of acyl peroxyl nitrates species [R(O)O_2_NO_2_ or peroxyacetyl nitrates (PANs)] [PANs→NO_2_ + R(O)O_2_], which increases concentrations of RO_2_ and HO*_X_* radicals via accelerated radical cycling. The increased RO*_X_* then accelerates the removal of NO*_X_*, resulting in reduced NO*_X_* concentration (fig. S5). Overall, the increase of RO*_X_* concentrations and the decrease of NO_2_ concentrations jointly lead to a decrease in φ and a shift toward a NO*_X_*-limited regime.

Furthermore, a higher temperature increases biogenic emissions of VOCs such as isoprene (fig. S6) and monoterpenes (*25*), promoting the production of secondary carbonyls (such as formaldehyde), which increases the primary production of RO*_X_* radicals (fig. S7) and thus reduces the lifetime of NO*_X_*, causing a further shift toward a NO*_X_*-limited regime. By accounting for the temperature dependence of chemistry and biogenic VOC emissions, the box model simulation shows a nonlinear dependence of φ on temperature (black lines in Fig. 2, A and B). Overall, both chemical processes and biogenic VOC emissions lead to a negative φ-temperature relationship, with associated slopes of −0.012° ± 0.004°C^−1^ and −0.015° ± 0.005°C^−1^, respectively, at temperatures above 20°C.

As a comparison with the box model simulation results, we use the GEOS-Chem model to investigate the underlying chemical mechanism behind the impact of temperature on O_3_ sensitivity in Beijing (see the “GEOS-Chem simulations” section). The model simulation can well reproduce the observed O_3_, NO*_X_* and VOC^R^ with average model deviations of <15% in Beijing (table S4). As shown in Fig. 2A and fig. S8, by accounting for the temperature dependence of chemistry and biogenic emissions (emissions of biogenic VOCs and soil NO*_X_*) in the GEOS-Chem simulation, the simulated relationship between φ and temperature is generally consistent with that calculated by the observation-based box model. The impact of temperature on soil NO*_X_* emissions plays a minor role in O_3_ sensitivity change compared with the impact of temperature on biogenic VOCs emissions, according to our model simulation. The temperature dependence of soil NO*_X_* emissions leads to an increase in φ of 0.03 from winter to summer, which is much smaller than the impact of the temperature dependence of biogenic VOC emissions (a reduction of 0.24 in φ).

According to the model simulations, the value of the slope between φ and temperature is rather small (about −0.0033°C^−1^) at temperature of <20°C, which is identified as a temperature-insensitive regime (Fig. 2A). The temperature-insensitive regime is attributed to the thermal stability of PAN (fig. S18) and the substantial decrease in biogenic VOC emissions (fig. S6) at temperatures below 20°C. The absolute value of the slope becomes larger at higher temperatures (>20°C), which is identified as a temperature-sensitive regime. Notably, the slope is again smaller at extremely high temperatures of >40°C (Fig. 2A), which arises because the thermal decomposition of PANs has less effect on chemistry and the biogenic emission of isoprene decreases due to biophysical high-temperature constraints at extremely high temperatures (*27*).

## DISCUSSION

### The impact of global warming on O_3_ sensitivity

Given the large effect of temperature change on O_3_ sensitivity and rising temperature due to global warming (*28*, *29*), the potential impact of global warming on O_3_ sensitivity is investigated through the GEOS-Chem model simulation. Here, we focus on the difference in O_3_ sensitivity between summer 2019 and a future scenario in summer 2100 characterized by the largest temperature increase [Shared Socioeconomic Pathway 5-8.5 (SSP5-8.5) scenario derived from Coupled Model Intercomparison Project Phase 6 (CMIP6) simulations] (*30*). Under the SSP5-8.5 scenario, summertime mean temperatures are projected to increase by 4° to 6°C from 2019 to 2100 across northern India, the North China Plain, and the southwestern United States. Notably, during the past 3 years (2023–2025), temperature anomalies of 4° to 6°C above the 2019 summertime mean have already occurred frequently in these regions on synoptic timescales, persisting for more than 10 days (*31*). Consequently, although SSP5-8.5 scenario may overestimate the long-term temperature increase by 2100, such extreme conditions are likely to occur under the exacerbated warming climate, particularly if future anthropogenic emission reductions are ineffective. Even if the monthly mean temperature in 2100 cannot reach up to such high values, the frequency and duration of extremely hot days are expected to increase substantially. In addition, selecting the SSP5-8.5 scenario for the analysis of O_3_ sensitivity is directly related to O_3_ pollution control strategy for extremely hot days, when O_3_ pollution is generally most severe.

**Fig. 3.**
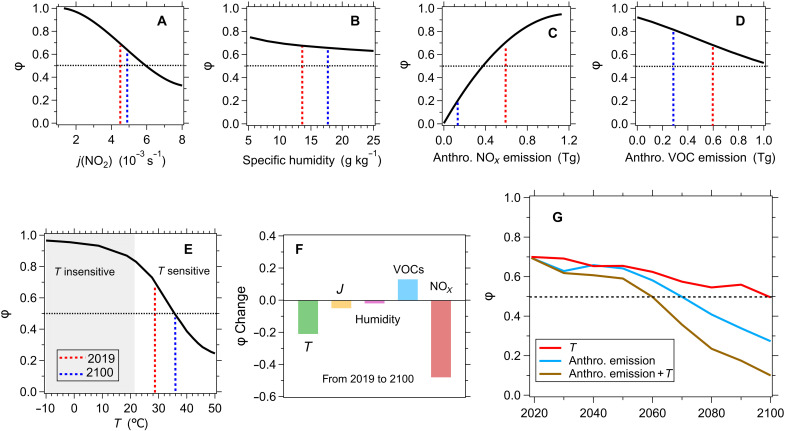
Drivers of O_3_ sensitivity change from 2019 to 2100 in July in Beijing. (**A** to **E**) GEOS-Chem model simulations showing the dependence of the monthly daytime average φ on key influential factors, including temperature (*T*), photolysis frequencies [e.g., *j*(NO_2_)], specific humidity, and anthropogenic emissions of NO*_X_* and VOCs. Red and blue dashed lines mark the corresponding values in 2019 and 2100, respectively. For each dependence, all conditions other than the investigated factor are fixed to the averages in 2019. The grey line represents the threshold value of 0.5. (**F**) The contributions of individual influential factors to the φ change from 2019 to 2100. (**G**) Temporal evolution of φ from 2019 to 2100 induced by temperature change (red line), changes in anthropogenic emissions (blue line), and changes in anthropogenic emissions and temperature (brown line). The temperature effect includes changes in reaction rate constants, biogenic emissions, and anthropogenic evaporative emissions. For all simulations, the influential factors were changed across the entire model domain (the North China Plain).

Figure 3 (A to F) shows the impacts of relevant influential factors on O_3_ sensitivity change from 2019 to 2100 in summer in Beijing (see the “Evaluating the dependence of φ on different influential factors” section). The temperature in Beijing is expected to increase from 28.5° to 35°C (*30*), which exactly falls into a temperature-sensitive regime. As a result, the temperature increase will cause a prominent decrease of φ by 0.21 (from 0.69 to 0.48), leading to a rapid shift toward the NO*_X_*-limited regime. Anthropogenic NO*_X_* emission abatement will lead to a decrease in φ by 0.47, whereas anthropogenic VOC emission abatement will lead to an increase in φ by 0.13. The enhancement in actinic flux and the increase in specific humidity play minor roles in φ compared with temperature change. Figure 3G shows the temporal evolution of φ from 2019 to 2100 with and without the impact of rising temperature considered. If the impact of rising temperature is not considered (blue line), then changes in anthropogenic emissions alone will shift O_3_ sensitivity from a VOC-limited regime to a NO*_X_*-limited regime in 2070. Additionally, considering the impact of rising temperature (brown line), this shift will occur earlier (in 2060), suggesting that rising temperature accelerates the shift of O_3_ sensitivity into a NO*_X_*-limited regime.

**Fig. 4.**
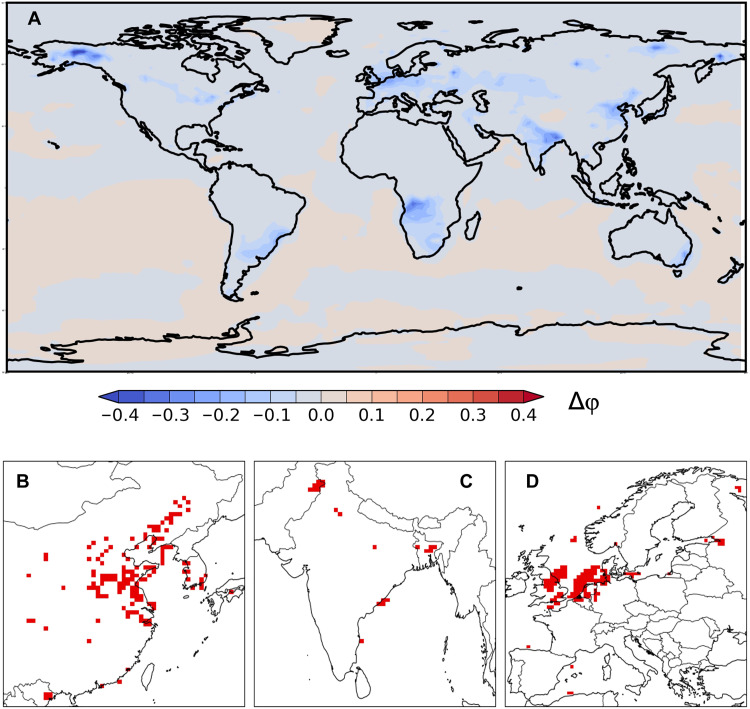
The impact of global warming on O_3_ sensitivity change from 2019 to 2100 across the world. GEOS-Chem simulated monthly daytime (8:00 to 18:00) average φ in July is used for analysis. (**A**) Global distribution of the change in φ (Δφ) from 2019 to 2100 due to the projected meteorological changes. (**B** to **D**) Red squares indicate the areas where O_3_ sensitivity shifts from a VOC-limited regime to a NO*_X_*-limited regime by 2100 in East Asia (B), South Asia (C), and Europe (D). Note that (A) is calculated using a 2° × 2.5° horizontal resolution but (B) to (D) are at 0.5° × 0.625°. Each red square in (B) to (D) represents a grid in the GEOS-Chem simulation with a resolution of 0.5° × 0.625°.

We further investigate the impact of future global warming on O_3_ sensitivity over the global scale by using the GEOS-Chem model (see the “Evaluating the impact of global warming on O_3_ sensitivity across the world” section). In the current scenario (2019), many cities in the North China Plain, northern India, and northwest of Europe are in a VOC-limited regime (fig. S10), which is consistent with previous studies (*8*, *32*, *33*). We simulate the worldwide change in summertime φ (Δφ) from 2019 to 2100 resulting from the meteorological changes (including temperature, humidity, solar radiation, winds, and boundary layer height) (as shown in Fig. 4A) and from both meteorological and anthropogenic emission changes (as depicted in fig. S15) under the SSP5-8.5 scenario. As shown in Fig. 4A, global warming will lead to a decrease in φ in many regions worldwide. In the hot-spot regions of O_3_ pollution such as the North China Plain (*34*) and northern India (*35*), φ will decrease by up to 0.3 (corresponding to 60%), indicating a prominent transition toward a NO*_X_*-limited regime. In addition, in Western Europe, southern Africa, southeast of Australia, and some coastal regions in America, φ also shows substantial decreases. As shown in Fig. 4 (B to D), O_3_ sensitivity in many cities over China, South Asia, and Europe will shift from a VOC-limited regime to a NO*_X_*-limited regime by 2100 due to climate change.

Our GEOS-Chem simulations suggest that, from 2019 to 2100, the overall contribution of temperature changes to Δφ is substantially larger than that of nontemperature meteorological changes (fig. S11). In this case, the rising temperature amplifies the benefit of NO*_X_* abatement in O_3_ mitigation under the warming climate, confirming that persistent NO*_X_* abatement will be favorable for long-term O_3_ pollution mitigation. Given that O_3_ is an important short-lived greenhouse gas (*36*), persistent NO*_X_* abatement for O_3_ pollution mitigation not only benefits for air quality improvement but also helps to alleviate global warming. The future variability in temperature will become an important source of uncertainty for accurate prediction of O_3_ sensitivity, which should be fully considered for the development of long-term O_3_ control strategies.

Moreover, climate change can influence O_3_ sensitivity regimes through meteorological changes beyond temperature, including changes in weather patterns, humidity, cloud cover, and the frequency and intensity of extremes. For example, more persistent summer heat waves can prolong photochemical episodes and may extend the duration of NO*_X_*-limited regimes through enhanced ROₓ radical production and increased biogenic VOC emissions. Warmer and wetter winters may also favor a shift toward NO*_X_*-limited regimes. Increased cloud cover can reduce photolysis frequencies, which may lead to a shift toward VOC-limited regimes. In addition, the covarying meteorological factors may locally modulate the magnitude and timing of regime changes and should be considered when interpreting effectiveness of future O_3_ mitigation strategies.

## MATERIALS AND METHODS

### Measurement data

Comprehensive measurements were conducted at urban sites in 2019 in Beijing (39.99° N, 116.31°E, PKUERS site) and Shanghai (31.17°N, 121.43°E), the two largest cities in China. In addition, we also conducted comprehensive measurements in summer 2014 in Wangdu, another city in the North China Plain. Beijing, Shanghai, and Wangdu suffer from severe O_3_ pollution and high oxidation capacity (*12*, *37*), serving as a useful reference for other Chinese cities that are still experiencing rapidly increasing O_3_ concentrations. Beijing and Wangdu are located within the North China Plain, a region that exhibits the highest O_3_ concentrations in China. Our GEOS-Chem simulations suggest that summertime daytime mean φ is 0.63 in Beijing and 0.51 in Wangdu, values that are close to the average across 24 cities in the North China Plain (0.60). Owing to their representativeness, these two cities have been the focus of numerous previous studies, including extensive field campaign measurements (*12*, *37*–*40*).

O_3_, nitric oxide (NO), nitrogen dioxide (NO_2_), VOCs, formaldehyde (HCHO), carbon monoxide (CO), sulfur dioxide (SO_2_), fine particulate matter (PM_2.5_), aerosol optical properties, and aerosol surface-area concentration (*S*_a_); photolysis frequencies of O_3_, NO_2_, NO_3_, HONO, and HCHO; and meteorological factors including temperature, relative humidity, wind speed, wind direction, and air pressure were measured during the study period. The measurement techniques are summarized in table S1, which have been well described in previous studies (*12*). Measurement data of O_3_, NO_2_, CO, and PM_2.5_ concentrations at other sites covering the whole cities were acquired from the public website of the China Ministry of Ecology and Environment (https://quotsoft.net/air/). OH, HO_2_, and RO_2_ radicals were measured by a laser-induced fluorescence technique in summer 2014 in Wangdu (*37*) and in winter 2016 in Beijing (*41*). Table S2 summarizes the measured species and measurement period of field campaigns used in this study.

Given that OH reactivity of VOCs (VOC^R^) is more related to O_3_ production compared to VOCs concentrations, we used VOC^R^ to quantify VOCs. VOC^R^ is defined as

$${\text{VOC}}^{R}=\sum_{i}^{n}k_{{\text{VOC}}_{i}}\left[{\text{VOC}}_{i}\right]$$ (2)

where $k_{{\text{VOC}}_{i}}$ represents the reaction rate constants of OH radicals with VOC species *i* and $\left[{\text{VOC}}_{i}\right]$ represents the concentration of VOC species *i*. Here, VOC^R^ includes both observed C_2_-C_10_ NMHCs and modeled oxidation intermediates.

### Observation-based photochemical box model

#### Model description

An observation-based photochemical box model is used to investigate the O_3_ sensitivity to precursors in typical Chinese cities (Beijing, Shanghai, Guangzhou, and Wangdu) in summer and winter. The box model applies the state-of-the-art chemical mechanism (MCM v3.3.1), which contains 5832 species and 17224 reactions (*42*, *43*), and can accurately simulate the radical source, cycling, and termination to understand the O_3_ formation. In this study, the model was initialized with 46 parent C_2_-C_10_ NMHCs, and 11030 reactions are active in the simulation. Model calculations are constrained to measured meteorological factors (i.e., photolysis frequencies, temperature, relative humidity, and air pressure), inorganic species (CO, SO_2_, and NO*_X_*), C_2_-C_10_ NMHCs with 46 species, and aerosol surface-area concentrations (*S*_a_). Aerosol surface-area concentrations were used to quantify heterogeneous uptake of HO_2_ radicals and reactive nitrogen species onto aerosols based on the method of Wang *et al.* (*12*). HO_2_ uptake coefficient used in the model is 0.08, which is derived from radical measurements and relevant budget analysis in the North China Plain (*44*). This approach takes the advantage of allowing detailed treatment of VOC oxidation chemistry, which is expected to be more accurate than lumped VOC mechanism such as Carbon Bond Mechanism (CBM), Statewide Air Pollution Research Center (SAPRC), and Regional Acid Deposition Model (RADM). The near-explicit model of radical sources, propagation, and amplification allow a robust analysis of the factors that govern the sensitivity of O_3_ production to its precursors. This approach has been successfully applied to study the nonlinear NO*_X_*-VOC-O_3_ relationship (*20*, *45*–*48*).

The model considers physical processes, including dilution, dry deposition, and transport. A first-order dilution parameter was used to represent all nonchemical loss of species through mixing to prevent the accumulation of unconstrained species within the model. The dilution rate was determined according to the diurnal variations in long-lived species (such as chloroform and methane) and boundary-layer height, according to the method reported by Edwards *et al.* (*20*). According to the model sensitivity test, a 50% change in dilution rate leads to variations of less than 15% in OH, HO_2_, RO_2_, and φ and less than 25% in key oxidation products such as HCHO, methyl vinyl ketone and methacrolein (MVK and MACR), and PAN. In addition to this dilution loss, the dry deposition rate for O_3_ was set as 0.42 cm s^−1^ in the daytime and 0.14 cm s^−1^ in the nighttime, as the deposition rate is known to decrease after sunset (*47*, *49*, *50*). Furthermore, the convection of O_3_ between near surface air and the residual layer aloft was considered in the box model, according to the method reported by Womack *et al.* (*47*). The model runs were performed in a time-dependent mode with a 1-hour resolution and 4-day spin-up. Model sensitivity test suggests that a 4-day spin-up produces the same modeled OH, HO_2_, and φ values as a 10-day spin-up, demonstrating that a 4-day spin-up is sufficient for the box model simulations. The box model used in this study is based on the Framework for 0-D Atmospheric Modeling (F0AM) (*51*). It integrates chemical rate equations with MATLAB’s ode15s solver, which is designed specifically for stiff systems. We simulated the scenarios in February and July in 2019, representing winter and summer respectively. In winter, periods with extremely high NO_*X*_ concentrations [>50 parts per billion (ppb)] were excluded from the analysis to avoid the influence of excessive NO_*X*_ titration.

As shown in fig. S1, the agreement between the simulated and observed oxidation products is excellent, with average model deviations of +12% in summer and −6% in winter for MVK and MACR and +18% in summer and +7% in winter for PAN. MVK and MACR are key oxidation products of isoprene, an important biogenic VOC. PAN is a crucial acyl peroxyl nitrates species [R(O)O_2_NO_2_, collectively referred to as PANs], and their relevant reactions play a key role in the temperature dependence of O_3_ sensitivity. The good performance of the model in simulating PAN suggests that the model can well capture the chemistry of the formation and decomposition of acyl peroxy nitrates. In addition, the box model can well simulate formaldehyde and acetaldehyde in Beijing, with discrepancies between observation and model simulation of less than 20%, which is consistent with a previous study (*12*). To consider primary emissions, we set the emission rates of formaldehyde and acetaldehyde in the model based on local emission inventories, specifically the Multi-resolution Emission Inventory for China (MEIC) (www.meicmodel.org/).

#### Construction of the O_3_ isopleth

The O_3_ isopleth as a function of NO*_X_* and VOCs was used here to assess the sensitivity of O_3_ to NO*_X_* and VOCs. The observed average diurnal variations of trace gaseous and meteorological factors in winter and summer were used to constrain the box model as the base scenario. To construct the O_3_ isopleth, we performed sensitivity simulations for each season by varying NO*_X_* concentrations and VOC^R^ from 0 to 200% (relative to the base scenario) with an interval of 10%. Note that model conditions other than VOCs and NO*_X_*, including photolysis frequencies, temperature, and humidity, kept the same as the base scenario. These sensitivity simulations provide sufficient data to illustrate the response of O_3_ to VOCs and NO*_X_* that could be used for diagnosing O_3_ sensitivity regimes.

#### *Evaluating the mechanism driving the temperature dependence of* φ

We used the box model to evaluate the mechanism driving the temperature dependence of O_3_ sensitivity. To evaluate the impact of temperature through altering reaction rate constants, in the model simulation, all conditions were fixed to summer 2019 in Beijing, and sensitivity tests were conducted by changing the reaction rate constant of the investigated reaction over a large temperature range (−10° to 50°C). To evaluate the impact of temperature through altering biogenic VOC emissions, in the model simulation, all conditions were fixed to summer 2019 in Beijing, and sensitivity tests were conducted by changing concentrations of isoprene and monoterpenes (α-pinene, β-pinene, and limonene) according to the observed temperature dependence of these biogenic VOCs (fig. S6). Notably, the OH reactivity of isoprene is higher than the OH reactivity of monoterpenes by a factor of 5 in Beijing and Shanghai, resulting in a dominant role of isoprene in determining the temperature dependence of φ.

Future reductions in NO_*X*_ emissions are expected to increase O_3_ concentrations in urban areas where the VOC-limited regime dominates, partly due to weakened NO titration of O_3_. The box model simulations indicate that a 20% reduction in NO_X_ leads to an increase of ~10 ppb in O_3_ in Beijing. We further examined the impact of a 20% and 50% NO_*X*_ reduction on the temperature dependence of O_3_ sensitivity in Beijing. As shown in fig. S12, a 20% reduction in NO*_X_* results in only a small change in the temperature dependence of φ, whereas a 50% reduction in NO_*X*_ has a larger effect.

#### The uncertainty of the model simulation

Uncertainty in the model simulations arises in part from uncertainties in key reaction rate constants relevant to O_3_ sensitivity, including those governing radical termination and cycling. Using the uncertainty factors listed in table S5, we conducted *n* model runs, where *n* equals the number of reactions considered. In each run, the rate constant of one reaction was perturbed by its uncertainty factor while all others were held constant. The overall model uncertainty was then quantified by propagating the errors from all perturbed reactions. Based on this approach, the uncertainty in the simulated φ is estimated to be 12%.

We have compared the key inorganic reaction rate constants in MCM v3.3.1 with the latest International Union of Pure and Applied Chemistry (IUPAC) recommendations (last accessed April 2026). The differences are mostly within 5%. For OH + NO → HONO, the difference is around 20%, resulting in only a slight change in φ, around 1.5%. The $k\left(\text{OH}+NO_{2}\to \text{HN}O_{3}\right)$ derived from MCM v3.3.1 is the same as that of the IUPAC evaluation. In addition, the MCM v3.3.1–derived $k\left(\text{OH}+NO_{2}\to \text{HN}O_{3}\right)$ increases by 18% from 274 K (winter) to 301 K (summer), whereas the IUPAC-recommended value shows a comparable increase of 17% over the same temperature range. This indicates that the temperature dependence of $k\left(\text{OH}+NO_{2}\to \text{HN}O_{3}\right)$ in MCM v3.3.1 is consistent with that in the latest IUPAC evaluation.

Similarly, model uncertainty also arises from uncertainties in measured trace gas concentrations and meteorological inputs, including photolysis frequencies (*J*), temperature, relative humidity, and air pressure. Using the uncertainty factors listed in table S1, we performed *n* model simulations (*n* equals the number of parameters considered). In each simulation, the value of the selected parameter was adjusted according to its uncertainty. The overall model uncertainty was then quantified through error propagation of uncertainties from all considered parameters. The resulting uncertainty in the simulated φ is ~24%.

Temperature changes can affect *j*(O^1^D) by altering the quantum yield and absorption cross section of this photolysis process. Our calculations indicate that a temperature change from summer to winter results in a ~3% decrease in *j*(O^1^D). In addition, the same temperature change leads to increases of 2 to 4% in the rate constants for reactions of O^1^D with H_2_O, O_2_, and N_2_. When these effects are considered together, the resulting change in φ due to the summer-to-winter temperature difference is less than 2%.

### GEOS-Chem simulations

#### Model description

To explore the impact of future changes in temperature on O_3_ sensitivity regime, we conducted simulations by applying the three-dimensional chemical transport model GEOS-Chem (version 13.3.3; https://geoschem.github.io/). The model fully simulates NO*_X_* + O*_X_* + CO + VOCs + HO*_X_* + Br + Cl + I + aerosols chemistry in the troposphere and stratosphere, and its good performance in simulating surface O_3_ in summer has been well evaluated in previous studies (*52*, *53*). In this study, we conducted the simulations over the globe. Global anthropogenic emissions used are from the Community Emission Data System inventory. In China, anthropogenic emission data are obtained from MEIC (www.meicmodel.org). Biogenic emissions are processed by the Model of Emissions of Gases and Aerosols from Nature (MEGAN) model version 2.1 according to the work of Guenther *et al.* (*54*). We used the assimilated meteorological data of Modern-Era Retrospective Analysis for Research and Applications, Version 2 (MERRA-2) to drive the model. The model simulations were conducted at a horizontal resolution of 2.5° longitude × 2.0° latitude with 47 layers for the global scale and at a horizontal resolution of 0.625° longitude × 0.5° latitude with 47 layers for regional scales including China, India, and Europe. For the key reactions governing O_3_ sensitivity—such as PANs ↔ RO_2_ + NO_2_, OH + NO_2_ → HNO_3_, HO_2_ + HO_2_ → H_2_O_2_, HO_2_ + NO → OH, isoprene oxidation by OH, and NO titration of O_3_—GEOS-Chem uses the same kinetics of reactions as MCM v3.3.1. To test whether the GEOS-Chem mechanism reproduces the temperature dependence of φ from MCM, we conducted parallel box model simulations using both mechanism under otherwise consistent conditions. As shown in fig. S13, the resulting temperature dependence of φ simulated with MCM v3.3.1 closely matches that obtained with the GEOS-Chem mechanism. This comparison supports the use of GEOS-Chem for capturing the dominant chemical controls on the temperature sensitivity of φ in this study.

We validated the model performance by comparing simulation results with observations in Beijing. As shown in table S4, the model simulation can well reproduce the observed O_3_, VOC^R^, and NO*_X_*, with average model deviations being smaller than ±15%. In addition, the model-simulated temperature dependence of isoprene is consistent with observed results (fig. S6). Our results show that the model overestimates surface O_3_ concentrations by 8 ppb in Beijing and by 5 ppb in the Beijing-Tianjin-Hebei region, comparable to the 6- to 8-ppb overestimation reported by Lu *et al.* (*55*). In Beijing, an overestimation in O_3_ of 8 ppb results in a 4.5% underestimation of φ (i.e., φ decreases from 0.65 to 0.62).

#### *Evaluating the dependence of* φ *on different influential factors*

Using the GEOS-Chem model, we investigated the driving forces governing O_3_ sensitivity by testing the responses of φ to the changes in relevant influential factors in July over Beijing. The investigated influential factors include temperature, photolysis frequencies, specific humidity, anthropogenic VOCs emissions, and anthropogenic NO*_X_* emissions. The simulation domain covers the North China Plain (fig. S14). For the simulation of each response, all conditions other than the investigated influential factor were fixed at July 2019. Over the simulation domain, each investigated influential factor was changed to span extremely low and extremely high values in real environments. As a result, the dependence of φ on each influential factor in Beijing was acquired as shown in Fig. 3. For the simulation of temperature dependence (orange line in Figs. 2A and 3E and fig. S8), the GEOS-Chem model accounts for the temperature dependence of chemistry and biogenic emissions including biogenic VOCs and soil NO*_X_*. In addition, we additionally test the impact of temperature dependence of evaporative anthropogenic VOC emissions including solvent use and transportation according to the parameterization method proposed by Wu *et al.* (*56*), which suggests a minor effect on φ compared to temperature dependence of chemistry and biogenic emissions (fig. S8). For all scenarios, the global background concentrations of O_3_, NO_*X*_, VOCs, and aerosols in July 2019 were used to drive the model simulations. Because O_3_ sensitivity is primarily governed by local photochemical reactions, using a fixed global background across scenarios is considered appropriate.

The diurnal variations of temperature, humidity, and photolysis frequencies are accounted for in the simulations. For the base scenario, hourly values of temperature and specific humidity from MERRA-2, along with hourly values of photolysis frequencies from the FAST-JX radiative transfer model, were used as model inputs for July 2019. To evaluate the impact of each influential factor on φ, temperature was varied over the range −30° to +30°C with 2°C increments, specific humidity was scaled by factors ranging from 0.8 to 2.0 with 0.2 increments, and photolysis frequencies of all photolysis reactions were varied by modifying the solar zenith angle in FAST-JX to represent higher or lower latitudes relative to the base domain. This approach ensures that diurnal variations remain consistent with the base scenario, despite changes in absolute values.

Notably, in Fig. 3 (A to E), temperature, photolysis frequencies and specific humidity correspond to the conditions in Beijing, whereas anthropogenic VOCs emissions and anthropogenic NO*_X_* emissions correspond to the conditions over the Beijing-Tianjin-Hebei region as the photochemistry in Beijing is largely influenced by emissions from Tianjin and Hebei. The data of these influential factors in future scenarios were acquired from model simulations of WCRP CMIP6 (https://esgf-data.dkrz.de/search/cmip6-dkrz/). The SSP5-8.5 scenario was used to test the potential influence of the largest increase of temperature.

According to CMIP6, near-surface photolysis frequencies in Beijing are projected to increase by ~9% from 2019 to 2100. This increase accounts for the effects of declining cloud cover and aerosol loading and leads to a reduction in φ of 0.05 (Fig. 3A). Specifically, cloud cover in Beijing is projected to decrease by 11.2%, contributing to a decrease in φ of 0.03.

#### Evaluating the impact of global warming on O_3_ sensitivity across the world

We used the GEOS-Chem model to simulate the change in O_3_ sensitivity from 2019 to 2100 across the world. For the base scenario, the simulations were run under conditions in July 2019. Based on the conditions in July 2019, we simulated the future scenario for July 2100 by using (1) meteorology in July 2100, (2) temperature alone in July 2100, (3) meteorology other than temperature in July 2100, (4) anthropogenic emissions in July 2100, (5) both meteorology and anthropogenic emissions in July 2100. As a result, the change in φ (Δφ) from 2019 to 2100 can be calculated. Δφ is shown in Fig. 4A for scenario 1, in fig. S11 for scenarios 2 to 4, and in fig. S15 for scenario 5. Table S7 summarizes the model input fields for GEOS-Chem simulations in 2019 and 2100.

Meteorological factors (including temperature, humidity, solar radiation, air pressure, wind fields, and boundary layer height) in 2100 were derived from CMIP6 (https://esgf-data.dkrz.de/search/cmip6-dkrz/). The SSP5-8.5 scenario was used in this study to test the potential influence of the largest increase of temperature. The outputs of 12 CMIP6 models (ACCESS-CM2, BCC-CSM2-MR, CanESM5, CESM2-WACCM, CMCC-ESM2, EC-Earth3-Veg-LR, FGOALS-f3-L, FGOALS-g3, GFDL-ESM4, NorESM2-MM, CESM2, and MIROC6) are averaged to quantify the changes in these meteorological factors from 2019 to 2100.

Running the GEOS-Chem model requires hourly meteorological data, whereas the CMIP6 provides only daily averages for the year 2100. The 2100 meteorological field was constructed by applying CMIP-derived 2100–2019 perturbations to the corresponding 2019 MERRA fields. Specifically, we calculated the daily mean difference between the CMIP simulations for 2100 and 2019 for a set of these variables (temperature, specific humidity, shortwave radiation, air pressure, and wind) and added the resulting perturbation to the corresponding hourly MERRA 2019 data, thereby generating a 2100 meteorological dataset based on the MERRA 2019 baseline (see text S3 for details). To quantify the impact of humidity, both specific humidity and relative humidity were used in the model simulation.

We regridded the CMIP6 meteorological data to match the MERRA-2 grid used in the GEOS-Chem model. Horizontally, bilinear interpolation was applied to align CMIP6 data with the MERRA-2 resolution grid. Vertically, CMIP6 data were regridded onto the 72 vertical layers of MERRA-2 based on the corresponding air pressure distributions. These 72 layers span the entire atmosphere, from the surface to the stratosphere.

The average of multiple models may not capture “weather,” particularly synoptic systems. We compare GEOS-Chem–simulated φ values for July 2100 under the average meteorology from multiple CMIP6 models and under the meteorology from a single model (CESM2) (fig. S16). The two scenarios correspond to scenario 5 and scenario 6 listed in table S7, which yield similar spatial distribution of φ and its change (fig. S16). This suggests that the key conclusions regarding the temperature dependence of φ remain valid.

The updated anthropogenic emissions in 2100 are taken from Gidden *et al.* (*57*), one of the most commonly used inventories for CMIP6. It is an emission inventory developed on the basis of SSP pathways with harmonized scenario trajectories and then downscaled/gridded for CMIP6 use. Because reliable uncertainty estimate is only possible for historical data, Gidden *et al.* (*57*) did not provide a quantitative estimate for future scenario emissions. However, they adopted a harmonization approach, where integrated assessment models (IAM) trajectories are systematically adjusted to match a common CMIP6 historical baseline in 2015, thereby reducing uncertainties in emission estimate. The anthropogenic emission inventories used for both 2019 and 2100 include anthropogenic emissions of CH_4_ and isoprene. Biogenic VOC emissions, including isoprene, monoterpenes, and sesquiterpenes, were calculated online using the MEGAN model, which accounts for the effects of meteorological variability and change. To quantify biomass burning emissions, the Quick Fire Emissions Dataset (QFED2) emission inventory for 2019 is used for the simulation in both 2019 and 2100 as a corresponding inventory for 2100 is not available.

In GEOS-Chem setup, the boundary layer height is taken from the meteorological datasets (MERRA-2), which is used to calculate the eddy-diffusivity profile. The GEOS-Chem adopted a nonlocal scheme formulated by Holtslag and Boville (*58*). The nonlocal scheme calculates the eddy-diffusivity profile based on the boundary layer height and a turbulent velocity scale, and it also accounts for nonlocal vertical transport of heat and moisture.

In our GEOS-Chem simulations, land use is held fixed at 2019 conditions for both the 2019 and 2100 scenarios. Therefore, direct impacts of land-use change on biogenic VOC emissions are not considered. On the other hand, the 2100 meteorological fields used to drive GEOS-Chem are taken from CMIP6 climate model output, in which land-use forcing is included as part of the climate scenario. Thus, impact of land-use change on the meteorology is implicitly considered. To provide context, we summarize CMIP6 land-use changes between 2019 and 2100 in fig. S17. This indicates that the largest changes occur primarily in high-latitude and tropical regions, whereas changes are comparatively smaller over several major O_3_-polluted regions. The small changes in land use are expected to have a limited impact on biogenic VOC emissions and O_3_ sensitivity in these regions. For example, CMIP6 suggests that forest cover increases by ~2% in China and decreases by ~3% in the United States. A MEGAN-based sensitivity test indicates that these changes would translate to a change of less than ~5% in biogenic VOC emissions, implying a relatively small impact on φ.

Figure S10 shows the spatial distribution of φ simulated by GEOS-Chem model in 2019 and 2100. As shown in fig. S10, under the current scenario (2019), many cities in the North China Plain, northern India, and northwest of Europe are in a VOC-limited regime (φ > 0.5). Most cities in North America have entered a NO*_X_*-limited regime (φ < 0.5). The forests and oceans are mostly in a NO*_X_*-limited regime (φ < 0.5). These results are generally consistent with previous studies (*8*, *10*, *32*, *33*, *59*, *60*).

Figure S11 shows the individual contributions of changes in temperature, nontemperature meteorological factors, and anthropogenic emissions to Δφ. The results indicate that the contribution of nontemperature meteorological changes to the change in φ from 2019 to 2100 is much smaller than that of temperature change. For example, over the North China Plain, temperature changes lead to a decrease in φ of ~0.20, whereas nontemperature meteorological changes result in a decrease of only about 0.04. The contribution of anthropogenic emission changes to Δφ varies across regions, leading to a decrease in φ in East Asia but an increase in most regions of Africa and Australia.

## References

[1] E. A. Ainsworth, C. R. Yendrek, S. Sitch, W. J. Collins, L. D. Emberson, The effects of tropospheric ozone on net primary productivity and implications for climate change. Annu. Rev. Plant Biol. 63, 637–661 (2012). 22404461 10.1146/annurev-arplant-042110-103829

[2] G. Mills, H. Pleijel, C. S. Malley, B. Sinha, O. R. Cooper, M. G. Schultz, H. S. Neufeld, D. Simpson, K. Sharps, Z. Feng, Tropospheric Ozone Assessment Report: Present-day tropospheric ozone distribution and trends relevant to vegetation. Elementa: Sci. Anthropol. 6, 47 (2018).

[3] P. S. Monks, A. T. Archibald, A. Colette, O. Cooper, M. Coyle, R. Derwent, D. Fowler, C. Granier, K. S. Law, G. E. Mills, D. S. Stevenson, O. Tarasova, V. Thouret, E. von Schneidemesser, R. Sommariva, O. Wild, M. L. Williams, Tropospheric ozone and its precursors from the urban to the global scale from air quality to short-lived climate forcer. Atmos. Chem. Phys. 15, 8889–8973 (2015).

[4] S. E. Pusede, A. L. Steiner, R. C. Cohen, Temperature and recent trends in the chemistry of continental surface ozone. Chem. Rev. 115, 3898–3918 (2015). 25950502 10.1021/cr5006815

[5] C. Hong, Q. Zhang, Y. Zhang, S. J. Davis, D. Tong, Y. Zheng, Z. Liu, D. Guan, K. He, H. J. Schellnhuber, Impacts of climate change on future air quality and human health in China. Proc. Natl. Acad. Sci. U.S.A. 116, 17193–17200 (2019). 31405979 10.1073/pnas.1812881116 PMC6717307

[6] P. Wang, Y. Yang, H. Li, L. Chen, R. Dang, D. Xue, B. Li, J. Tang, L. R. Leung, H. Liao, North China Plain as a hot spot of ozone pollution exacerbated by extreme high temperatures. Atmos. Chem. Phys. 22, 4705–4719 (2022).

[7] L. I. Kleinman, Low and high NO_x_ tropospheric photochemistry. J. Geophys. Res.-Atmos. 99, 16831–16838 (1994).

[8] P. D. Ivatt, M. J. Evans, A. C. Lewis, Suppression of surface ozone by an aerosol-inhibited photochemical ozone regime. Nat. Geosci. 15, 536–540 (2022).

[9] S. Sillman, The relation between ozone, NO*_x_* and hydrocarbons in urban and polluted rural environments. Atmos. Environ. 33, 1821–1845 (1999).

[10] J. L. Laughner, R. C. Cohen, Direct observation of changing NO*_x_* lifetime in North American cities. Science 366, 723–727 (2019). 31699933 10.1126/science.aax6832 PMC7301961

[11] Q. Zhu, J. L. Laughner, R. C. Cohen, Estimate of OH trends over one decade in North American cities. Proc. Natl. Acad. Sci. U.S.A. 119, e2117399119 (2022). 35412909 10.1073/pnas.2117399119 PMC9169711

[12] W. Wang, X. Li, Y. Cheng, D. D. Parrish, R. Ni, Z. Tan, Y. Liu, S. Lu, Y. Wu, S. Chen, K. Lu, M. Hu, L. Zeng, M. Shao, C. Huang, X. Tian, K. M. Leung, L. Chen, M. Fan, Q. Zhang, F. Rohrer, A. Wahner, U. Pöschl, H. Su, Y. Zhang, Ozone pollution mitigation strategy informed by long-term trends of atmospheric oxidation capacity. Nat. Geosci. 17, 20–25 (2024).

[13] Y. Wang, Y. Zhao, Y. Liu, Y. Jiang, B. Zheng, J. Xing, Y. Liu, S. Wang, C. P. Nielsen, Sustained emission reductions have restrained the ozone pollution over China. Nat. Geosci. 16, 967–974 (2023).

[14] J. Coates, K. A. Mar, N. Ojha, T. M. Butler, The influence of temperature on ozone production under varying NO*_x_* conditions – A modelling study. Atmos. Chem. Phys. 16, 11601–11615 (2016).

[15] M. Li, X. Huang, D. Yan, S. Lai, Z. Zhang, L. Zhu, Y. Lu, X. Jiang, N. Wang, T. Wang, Y. Song, A. Ding, Coping with the concurrent heatwaves and ozone extremes in China under a warming climate. Sci. Bull. 69, 2938–2947 (2024). 10.1016/j.scib.2024.05.034 38944635

[16] G. Churkina, F. Kuik, B. Bonn, A. Lauer, R. Grote, K. Tomiak, T. M. Butler, Effect of VOC emissions from vegetation on air quality in Berlin during a heatwave. Environ. Sci. Technol. 51, 6120–6130 (2017). 28513175 10.1021/acs.est.6b06514

[17] M. Lin, L. W. Horowitz, Y. Xie, F. Paulot, S. Malyshev, E. Shevliakova, A. Finco, G. Gerosa, D. Kubistin, K. Pilegaard, Vegetation feedbacks during drought exacerbate ozone air pollution extremes in Europe. Nat. Clim. Change. 10, 444–451 (2020).

[18] M. Qin, Y. She, M. Wang, H. Wang, Y. Chang, Z. Tan, J. An, J. Huang, Z. Yuan, J. Lu, Q. Wang, C. Liu, Z. Liu, X. Xie, J. Li, H. Liao, H. O. T. Pye, C. Huang, S. Guo, M. Hu, Y. Zhang, D. J. Jacob, J. Hu, Increased urban ozone in heat waves due to temperature-induced emissions of anthropogenic volatile organic compounds. Nat. Geosci. 18, 50–56 (2025). 40547950 10.1038/s41561-024-01608-w PMC12180938

[19] M. E. Jenkin, J. C. Young, A. R. Rickard, The MCM v3.3.1 degradation scheme for isoprene. Atmos. Chem. Phys. 15, 11433–11459 (2015).

[20] P. M. Edwards, S. S. Brown, J. M. Roberts, R. Ahmadov, R. M. Banta, J. A. deGouw, W. P. Dubé, R. A. Field, J. H. Flynn, J. B. Gilman, M. Graus, D. Helmig, A. Koss, A. O. Langford, B. L. Lefer, B. M. Lerner, R. Li, S.-M. Li, S. A. McKeen, S. M. Murphy, D. D. Parrish, C. J. Senff, J. Soltis, J. Stutz, C. Sweeney, C. R. Thompson, M. K. Trainer, C. Tsai, P. R. Veres, R. A. Washenfelder, C. Warneke, R. J. Wild, C. J. Young, B. Yuan, R. Zamora, High winter ozone pollution from carbonyl photolysis in an oil and gas basin. Nature 514, 351–354 (2014). 25274311 10.1038/nature13767

[21] M. Kang, J. Zhang, H. Zhang, Q. Ying, On the relevancy of observed ozone increase during COVID-19 lockdown to summertime ozone and PM2.5 control policies in China. Environ. Sci. Technol. Lett. 8, 289–294 (2021). 37566348 10.1021/acs.estlett.1c00036

[22] S. Sillman, D. He, Some theoretical results concerning O_3_-NO*_x_*-VOC chemistry and NO*_x_*-VOC indicators. J. Geophys. Res.-Atmos. 107, ACH 26–21–ACH 26–15 (2002).

[23] A. Hofzumahaus, F. Rohrer, K. Lu, B. Bohn, T. Brauers, C.-C. Chang, H. Fuchs, F. Holland, K. Kita, Y. Kondo, Amplified trace gas removal in the troposphere. Science 324, 1702–1704 (2009). 19498111 10.1126/science.1164566

[24] F. Rohrer, K. Lu, A. Hofzumahaus, B. Bohn, T. Brauers, C.-C. Chang, H. Fuchs, R. Häseler, F. Holland, M. Hu, Maximum efficiency in the hydroxyl-radical-based self-cleansing of the troposphere. Nat. Geosci. 7, 559–563 (2014).

[25] E. Y. Pfannerstill, C. Arata, Q. Zhu, B. C. Schulze, R. Ward, R. Woods, C. Harkins, R. H. Schwantes, J. H. Seinfeld, A. Bucholtz, R. C. Cohen, A. H. Goldstein, Temperature-dependent emissions dominate aerosol and ozone formation in Los Angeles. Science 384, 1324–1329 (2024). 38900887 10.1126/science.adg8204

[26] J. Burkholder, S. Sander, J. Abbatt, J. Barker, C. Cappa, J. Crounse, T. Dibble, R. Huie, C. Kolb, M. Kurylo, “Chemical kinetics and photochemical data for use in atmospheric studies; evaluation number 19” (Jet Propulsion Laboratory, National Aeronautics and Space, 2020).

[27] A. L. Steiner, A. J. Davis, S. Sillman, R. C. Owen, A. M. Michalak, A. M. Fiore, Observed suppression of ozone formation at extremely high temperatures due to chemical and biophysical feedbacks. Proc. Natl. Acad. Sci. U.S.A. 107, 19685–19690 (2010). 21041679 10.1073/pnas.1008336107 PMC2993403

[28] E. Fischer, S. Sippel, R. Knutti, Increasing probability of record-shattering climate extremes. Nat. Clim. Change. 11, 689–695 (2021). 10.1038/s41558-021-01092-9 PMC7617090 39650282

[29] K. Thirumalai, P. N. DiNezio, Y. Okumura, C. Deser, Extreme temperatures in Southeast Asia caused by El Nino and worsened by global warming. Nat. Commun. 8, 15531 (2017). 28585927 10.1038/ncomms15531 PMC5467164

[30] T. F. Stocker, D. Qin, G.-K. Plattner, M. M. Tignor, S. K. Allen, J. Boschung, A. Nauels, Y. Xia, V. Bex, P. M. Midgley, *Climate Change 2013 – The Physical Science Basis: Working Group I Contribution to the Fifth Assessment Report of the Intergovernmental Panel on Climate Change* (Cambridge Univ. Press, 2014).

[31] J. Coleman, Chance of heatwaves in India rising with climate change. Nature 10.1038/d41586-024-01577-5 (2024). 38811783

[32] M. Beekmann, R. Vautard, A modelling study of photochemical regimes over Europe: Robustness and variability. Atmos. Chem. Phys. 10, 10067–10084 (2010).

[33] T. Wang, L. Xue, Z. Feng, J. Dai, Y. Zhang, Y. Tan, Ground-level ozone pollution in China: A synthesis of recent findings on influencing factors and impacts. Environ. Res. Lett. 17, 063003 (2022).

[34] X. Lu, J. Y. Hong, L. Zhang, O. R. Cooper, M. G. Schultz, X. B. Xu, T. Wang, M. Gao, Y. H. Zhao, Y. H. Zhang, Severe surface ozone pollution in China: A global perspective. Environ. Sci. Technol. Lett. 5, 487–494 (2018).

[35] M. Pommier, H. Fagerli, M. Gauss, D. Simpson, S. Sharma, V. Sinha, S. D. Ghude, O. Landgren, A. Nyiri, P. Wind, Impact of regional climate change and future emission scenarios on surface O_3_ and PM_2.5_ over India. Atmos. Chem. Phys. 18, 103–127 (2018).

[36] IPCC, *Climate Change 2021 – The Physical Science Basis: Working Group I Contribution to the Sixth Assessment Report of the Intergovernmental Panel on Climate Change* (Cambridge Univ. Press, 2023).

[37] Z. Tan, H. Fuchs, K. Lu, A. Hofzumahaus, B. Bohn, S. Broch, H. Dong, S. Gomm, R. Häseler, L. He, F. Holland, X. Li, Y. Liu, S. Lu, F. Rohrer, M. Shao, B. Wang, M. Wang, Y. Wu, L. Zeng, Y. Zhang, A. Wahner, Y. Zhang, Radical chemistry at a rural site (Wangdu) in the North China Plain: Observation and model calculations of OH, HO_2_ and RO_2_ radicals. Atmos. Chem. Phys. 17, 663–690 (2017).

[38] H. Fuchs, Z. Tan, K. Lu, B. Bohn, S. Broch, S. S. Brown, H. Dong, S. Gomm, R. Häseler, L. He, A. Hofzumahaus, F. Holland, X. Li, Y. Liu, S. Lu, K. E. Min, F. Rohrer, M. Shao, B. Wang, M. Wang, Y. Wu, L. Zeng, Y. Zhang, A. Wahner, Y. Zhang, OH reactivity at a rural site (Wangdu) in the North China Plain: Contributions from OH reactants and experimental OH budget. Atmos. Chem. Phys. 17, 645–661 (2017).

[39] C. Xue, C. Ye, K. Lu, P. Liu, C. Zhang, H. Su, F. Bao, Y. Cheng, W. Wang, Y. Liu, V. Catoire, Z. Ma, X. Zhao, Y. Song, X. Ma, M. R. McGillen, A. Mellouki, Y. Mu, Y. Zhang, Reducing soil-emitted nitrous acid as a feasible strategy for tackling ozone pollution. Environ. Sci. Technol. 58, 9227–9235 (2024). 38751196 10.1021/acs.est.4c01070 PMC11137860

[40] K. Lu, Y. Zhang, H. Su, T. Brauers, C. C. Chou, A. Hofzumahaus, S. C. Liu, K. Kita, Y. Kondo, M. Shao, A. Wahner, J. Wang, X. Wang, T. Zhu, Oxidant (O_3_ + NO_2_) production processes and formation regimes in Beijing. J. Geophys. Res.-Atmos. 115, D07303 (2010).

[41] Z. Tan, F. Rohrer, K. Lu, X. Ma, B. Bohn, S. Broch, H. Dong, H. Fuchs, G. I. Gkatzelis, A. Hofzumahaus, F. Holland, X. Li, Y. Liu, Y. Liu, A. Novelli, M. Shao, H. Wang, Y. Wu, L. Zeng, M. Hu, A. Kiendler-Scharr, A. Wahner, Y. Zhang, Wintertime photochemistry in Beijing: Observations of RO*_x_* radical concentrations in the North China Plain during the BEST-ONE campaign. Atmos. Chem. Phys. 18, 12391–12411 (2018).

[42] M. Jenkin, S. Saunders, V. Wagner, M. Pilling, Protocol for the development of the Master Chemical Mechanism, MCM v3 (Part B): Tropospheric degradation of aromatic volatile organic compounds. Atmos. Chem. Phys. 3, 181–193 (2003).

[43] S. M. Saunders, M. E. Jenkin, R. Derwent, M. Pilling, Protocol for the development of the Master Chemical Mechanism, MCM v3 (Part A): Tropospheric degradation of non-aromatic volatile organic compounds. Atmos. Chem. Phys. 3, 161–180 (2003).

[44] Z. Tan, A. Hofzumahaus, K. Lu, S. S. Brown, F. Holland, L. G. Huey, A. Kiendler-Scharr, X. Li, X. Liu, N. Ma, No evidence for a significant impact of heterogeneous chemistry on radical concentrations in the North China plain in summer 2014. Environ. Sci. Technol. 54, 5973–5979 (2020). 32343120 10.1021/acs.est.0c00525 PMC7240937

[45] W. P. Carter, J. H. Seinfeld, Winter ozone formation and VOC incremental reactivities in the Upper Green River Basin of Wyoming. Atmos. Environ. 50, 255–266 (2012).

[46] Y. Chen, G. Beig, S. Archer-Nicholls, W. Drysdale, W. J. F. Acton, D. Lowe, B. Nelson, J. Lee, L. Ran, Y. Wang, Avoiding high ozone pollution in Delhi, India. Faraday Discuss. 226, 502–514 (2021). 33244555 10.1039/d0fd00079e

[47] C. C. Womack, E. E. McDuffie, P. M. Edwards, R. Bares, J. A. de Gouw, K. S. Docherty, W. P. Dubé, D. L. Fibiger, A. Franchin, J. B. Gilman, L. Goldberger, B. H. Lee, J. C. Lin, R. Long, A. M. Middlebrook, D. B. Millet, A. Moravek, J. G. Murphy, P. K. Quinn, T. P. Riedel, J. M. Roberts, J. A. Thornton, L. C. Valin, P. R. Veres, A. R. Whitehill, R. J. Wild, C. Warneke, B. Yuan, M. Baasandorj, S. S. Brown, An odd oxygen framework for wintertime ammonium nitrate aerosol pollution in urban areas: NO*_x_* and VOC control as mitigation strategies. Geophys. Res. Lett. 46, 4971–4979 (2019).

[48] M. Shao, W. Wang, B. Yuan, D. D. Parrish, X. Li, K. Lu, L. Wu, X. Wang, Z. Mo, S. Yang, Quantifying the role of PM2.5 dropping in variations of ground-level ozone: Inter-comparison between Beijing and Los Angeles. Sci. Total Environ. 788, 147712 (2021). 34134364 10.1016/j.scitotenv.2021.147712

[49] L. Zhang, J. R. Brook, R. Vet, A revised parameterization for gaseous dry deposition in air-quality models. Atmos. Chem. Phys. 3, 2067–2082 (2003).

[50] S. Yang, B. Yuan, Y. Peng, S. Huang, W. Chen, W. Hu, C. Pei, J. Zhou, D. D. Parrish, W. Wang, X. He, C. Cheng, X. B. Li, X. Yang, Y. Song, H. Wang, J. Qi, B. Wang, C. Wang, C. Wang, Z. Wang, T. Li, E. Zheng, S. Wang, C. Wu, M. Cai, C. Ye, W. Song, P. Cheng, D. Chen, X. Wang, Z. Zhang, X. Wang, J. Zheng, M. Shao, The formation and mitigation of nitrate pollution: Comparison between urban and suburban environments. Atmos. Chem. Phys. 22, 4539–4556 (2022).

[51] G. M. Wolfe, M. R. Marvin, S. J. Roberts, K. R. Travis, J. Liao, The Framework for 0-D Atmospheric Modeling (F0AM) v3.1. Geosci. Model Dev. 9, 3309–3319 (2016).

[52] R. Ni, J. Lin, Y. Yan, W. Lin, Foreign and domestic contributions to springtime ozone over China. Atmos. Chem. Phys. 18, 11447–11469 (2018).

[53] K. Li, D. J. Jacob, H. Liao, L. Shen, Q. Zhang, K. H. Bates, Anthropogenic drivers of 2013–2017 trends in summer surface ozone in China. Proc. Natl. Acad. Sci. U.S.A. 116, 422–427 (2019). 30598435 10.1073/pnas.1812168116 PMC6329973

[54] A. B. Guenther, X. Jiang, C. L. Heald, T. Sakulyanontvittaya, T. Duhl, L. K. Emmons, X. Wang, The model of emissions of gases and aerosols from nature version 2.1 (MEGAN2.1): An extended and updated framework for modeling biogenic emissions. Geosci. Model Dev. 5, 1471–1492 (2012).

[55] X. Lu, Y. Liu, J. Su, X. Weng, T. Ansari, Y. Zhang, G. He, Y. Zhu, H. Wang, G. Zeng, J. Li, C. He, S. Li, T. Amnuaylojaroen, T. Butler, Q. Fan, S. Fan, G. L. Forster, M. Gao, J. Hu, Y. Kanaya, M. T. Latif, K. Lu, P. Nédélec, P. Nowack, B. Sauvage, X. Xu, L. Zhang, K. Li, J. H. Koo, T. Nagashima, Tropospheric ozone trends and attributions over East and Southeast Asia in 1995–2019: An integrated assessment using statistical methods, machine learning models, and multiple chemical transport models. Atmos. Chem. Phys. 25, 7991–8028 (2025).

[56] W. Wu, T.-M. Fu, S. R. Arnold, D. V. Spracklen, A. Zhang, W. Tao, X. Wang, Y. Hou, J. Mo, J. Chen, Y. Li, X. Feng, H. Lin, Z. Huang, J. Zheng, H. Shen, L. Zhu, C. Wang, J. Ye, X. Yang, Temperature-dependent evaporative anthropogenic VOC emissions significantly exacerbate regional ozone pollution. Environ. Sci. Technol. 58, 5430–5441 (2024). 38471097 10.1021/acs.est.3c09122 PMC10976895

[57] M. J. Gidden, K. Riahi, S. J. Smith, S. Fujimori, G. Luderer, E. Kriegler, D. P. van Vuuren, M. van den Berg, L. Feng, D. Klein, K. Calvin, J. C. Doelman, S. Frank, O. Fricko, M. Harmsen, T. Hasegawa, P. Havlik, J. Hilaire, R. Hoesly, J. Horing, A. Popp, E. Stehfest, K. Takahashi, Global emissions pathways under different socioeconomic scenarios for use in CMIP6: A dataset of harmonized emissions trajectories through the end of the century. Geosci. Model Dev. 12, 1443–1475 (2019).

[58] A. A. M. Holtslag, B. A. Boville, Local versus nonlocal boundary-layer diffusion in a global climate model. J. Climate 6, 1825–1842 (1993).

[59] S. Sillman, J. A. Logan, S. C. Wofsy, The sensitivity of ozone to nitrogen oxides and hydrocarbons in regional ozone episodes. J. Geophys. Res.-Atmos. 95, 1837–1851 (1990).

[60] G. S. Tonnesen, R. L. Dennis, Analysis of radical propagation efficiency to assess ozone sensitivity to hydrocarbons and NO*_x_*: 2. Long-lived species as indicators of ozone concentration sensitivity. J. Geophys. Res.-Atmos. 105, 9227–9241 (2000).

