# Supplementary material for: Global warming makes nitrogen oxide abatement key to ozone pollution mitigation
Source: Sci Adv. 2026 Jul 17;12(29):eaea4124. doi: 10.1126/sciadv.aea4124 (PMC13378576; doi:10.1126/sciadv.aea4124)
Supplement: Supplementary file 1 — Text S1 to S3 Figs. S1 to S18 Tables S1 to S7 [file sciadv.aea4124_sm.pdf]

Science Advances
AAAS

Supplementary Materials for

Global warming makes nitrogen oxide abatement key to ozone pollution mitigation

Wenjie Wang *et al.*

Corresponding author: Yafang Cheng, yafang.cheng@mpic.de; Hang Su, suhang@mail.iap.ac.cn

Sci. Adv. **12**, eaea4124 (2026)
DOI: 10.1126/sciadv.aea4124

This PDF file includes:

Text S1 to S3
Figs. S1 to S18
Tables S1 to S7

Text S1 The definition and diagnosis of O_3 sensitivity regimes

In this study, we adopted the traditional two-regime scheme (VOC-limited and NO_X-limited), without additionally splitting out a transitional regime. In the framework of the two-regime scheme, NO_X emission control is more effective in reducing O_3 than VOC emission control in the NO_X-limited regime, while reducing VOC emissions is more effective in reducing O_3 than NO_X emission control in the VOC-limited regime. As a result, the two-regime scheme can already provide a clear insight into the relative effectiveness of VOC and NO_X emission control in reducing O_3 formation, and inform which of the two precursors should be controlled in priority (*8, 22*). Some studies additionally defined a transitional regime between VOC-limited and NO_X-limited regimes, in which O_3 formation can be sensitive to both NO_X and VOC controls. The transitional regime has diverse definitions in different studies and lacks of a specific indicator and threshold, which makes it difficult to diagnose the transitional regime clearly. Even in the transitional regime, O_3 production still exhibits more VOC-limited or more NO_X-limited. Therefore, we adopted the traditional two-regime scheme for analysis here.

According to the study of Sillman et al (*22*), the split of O_3 sensitivity regimes depends on the relative rate of formation of peroxides (via HO_2-HO_2 and HO_2-RO_2 reactions) relative to nitric acid formation (via OH + NO_2) (*22*). They also emphasized that the split of O_3 sensitivity is associated with formation of nitric acid but is not affected by formation of PANs (*22, 59, 60*). As a result, the ratio of OH+NO_2 reaction

rate to the total reaction rate of OH+NO_2, HO_2-HO_2 and HO_2-RO_2, defined as φ, is used to diagnose O_3 sensitivity regimes in this study. The definition of φ does not include the reaction rate of PANs formation. The threshold value of φ that distinguishes between VOC-limited and NO_X-limited regimes is 0.5. A recent study also adopted the relative importance between formation of peroxides and nitric acid formation to distinguish O_3 sensitivity regimes worldwide (*8*).

Text S2 The effect of PAN chemistry on temperature dependence of O_3 sensitivity

The classical definition of radical termination includes reactions such as $\mathrm{OH} + \mathrm{NO_2}$, $\mathrm{RO_2} + \mathrm{RO_2}$, and heterogeneous uptake of $\mathrm{HO_2}$, which represent irreversible sinks of radicals. In contrast, PANs are not strictly a permanent radical sink, but rather a reversible sink that can release radicals upon decomposition. As shown in Fig. R1 (Fig. S18 in the SI of our manuscript), at relatively low temperatures (T < 20 °C), PANs have a long lifetime and effectively act as an effective sink of radicals over relevant timescales, leading to a temperature-insensitive regime of O_3–precursor sensitivity. In this regime, PAN formation can be viewed as functionally analogous to a radical termination step. In contrast, at higher temperatures (T > 20 °C), the lifetime of PANs decreases substantially due to rapid thermal decomposition. Under these conditions, PANs don't behave like an irreversible radical sink but instead acts as a temperature-dependent reservoir. Therefore, PAN chemistry influences O_3–precursor sensitivity by controlling the radical termination efficiency in a temperature-dependent manner.

Text S3 The uncertainty due to averaging wind fields from multiple CMIP models

Averaging wind fields from multiple CMIP models may introduce mass conservation inconsistencies. To assess this effect, for the GEOS-Chem 2100 scenarios we performed three sets of simulations using: (1) 2100 temperature only, based on the CMIP6 multi-model mean (Fig. S11 A); (2) the constructed 2100 meteorology, based on a single CMIP model (CESM2) (Fig.S16 B); (3) the constructed 2100 meteorology, based on the CMIP6 multi-model mean (Fig. S16 A). Notably, scenarios (1) and (2) do not suffer from mass conservation inconsistencies.

To assess the effect of potential mass conservation inconsistencies in scenario (3) (induced by averaging wind fields from multiple models), we compared it with scenario (2). As shown in Fig. S16, the resulting spatial patterns and temperature dependence of φ are highly consistent with those from our baseline simulations. The global mean difference in φ is approximately 3% and the relevant conclusions remain unchanged.

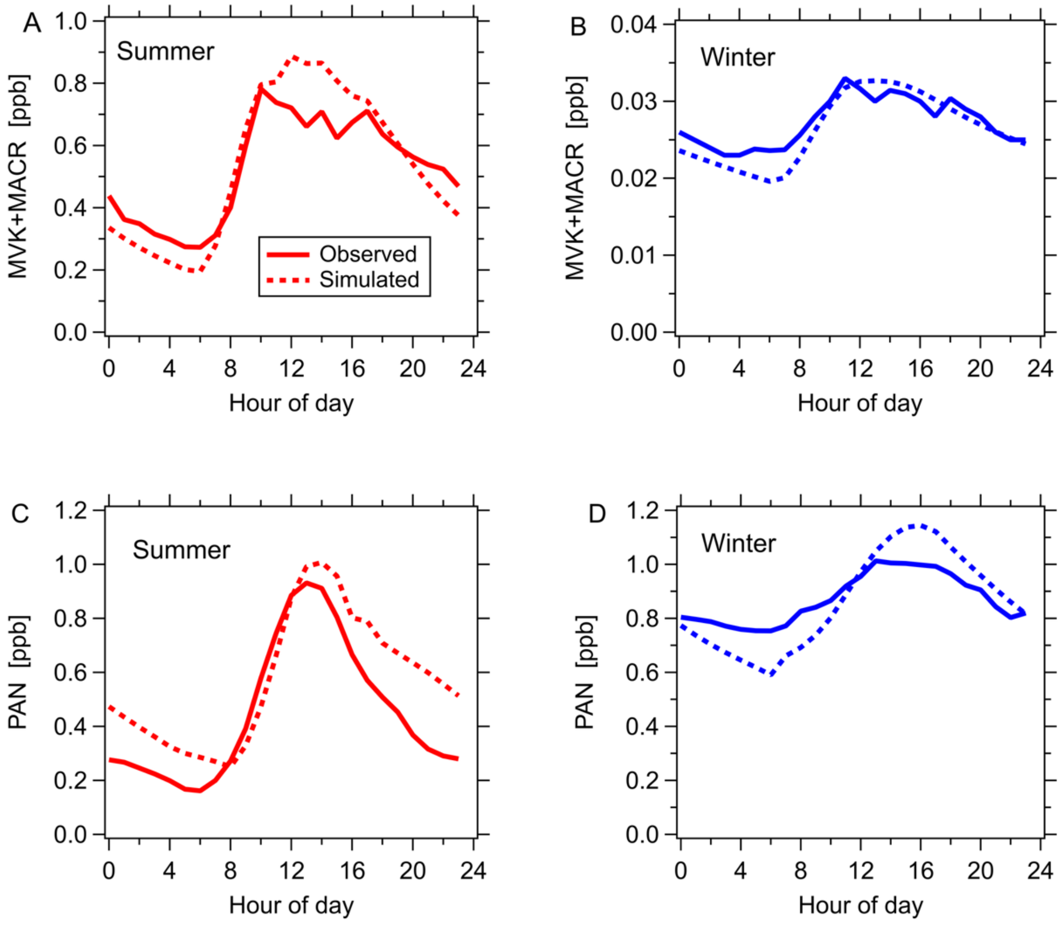

76 **Figure S1. Observed and box model-simulated MVK+MACR and PAN in**
77 **summer and winter.**

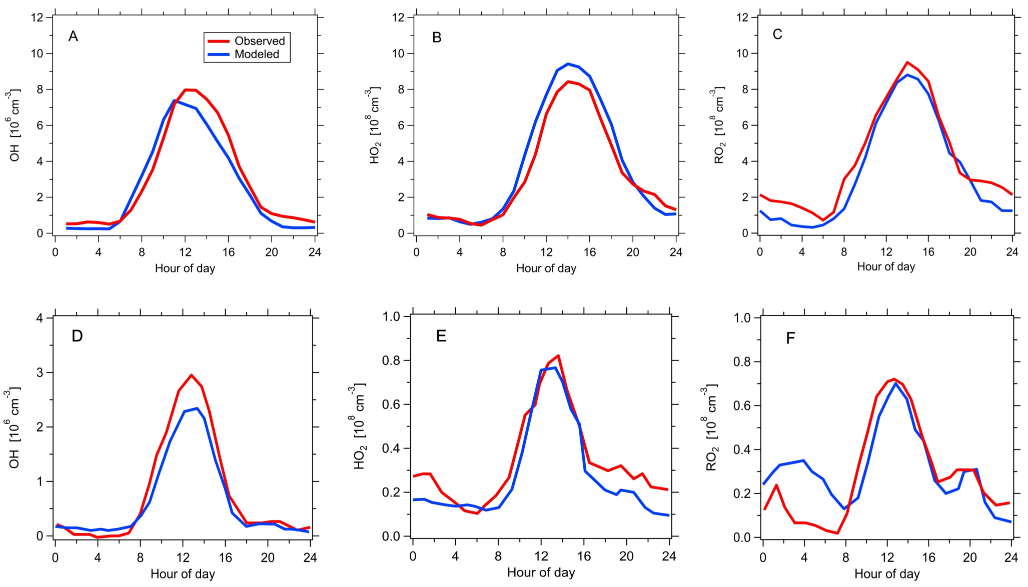

Figure S2. The comparison of measurements (red line) and box model simulations (blue line) for OH, HO_2 and RO_2 radicals in summer 2014 in Wangdu (A-C) and in winter 2016 in Beijing (D-F). The RO_X radicals were measured by a laser-induced fluorescence (LIF) technique.

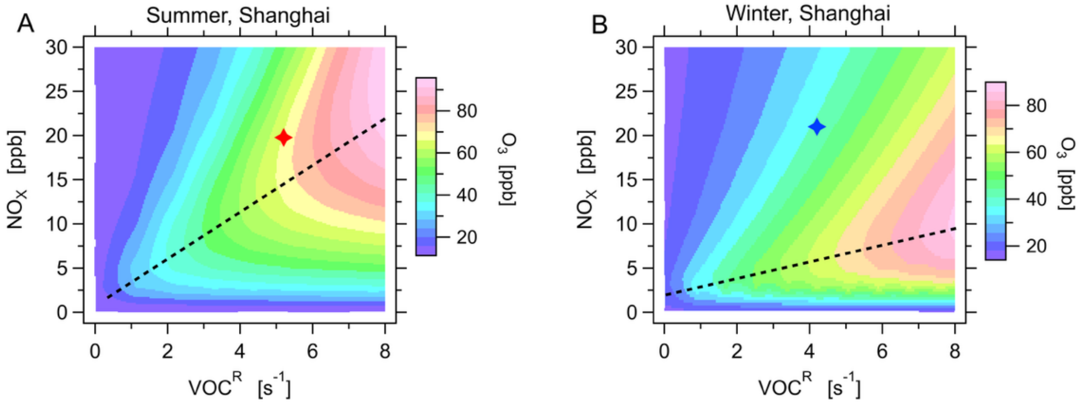

Figure S3. The contrast of O_3 sensitivity between summer and winter in 2019 in Shanghai. Box model-simulated isopleth diagrams for O_3 as a function of NO_X and VOCs in summer and winter, respectively. VOCs are quantified by OH reactivity of VOCs (VOC^R) (Supplementary S1). The averages of NO_X and VOC^R in summer (red symbol) and winter (blue symbol) are marked on the isopleth diagrams. The dashed lines indicate the ridge lines.

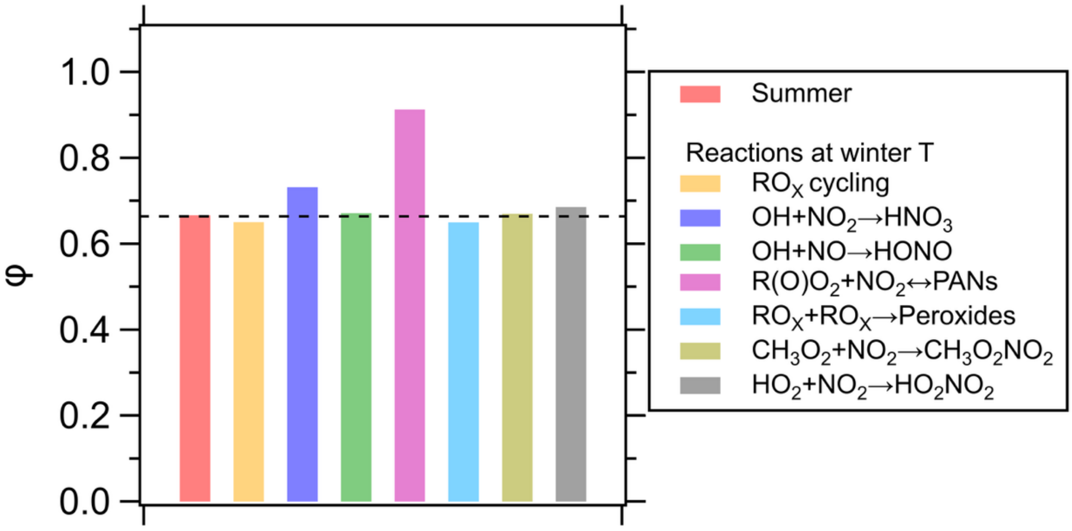

Figure S4. Box model-simulated φ in Beijing. The summer scenario is taken as the base scenario, and sensitivity test is conducted by adjusting the reaction rate constant of individual reactions to the wintertime temperature. The dotted line is the ozone sensitivity for the base scenario.

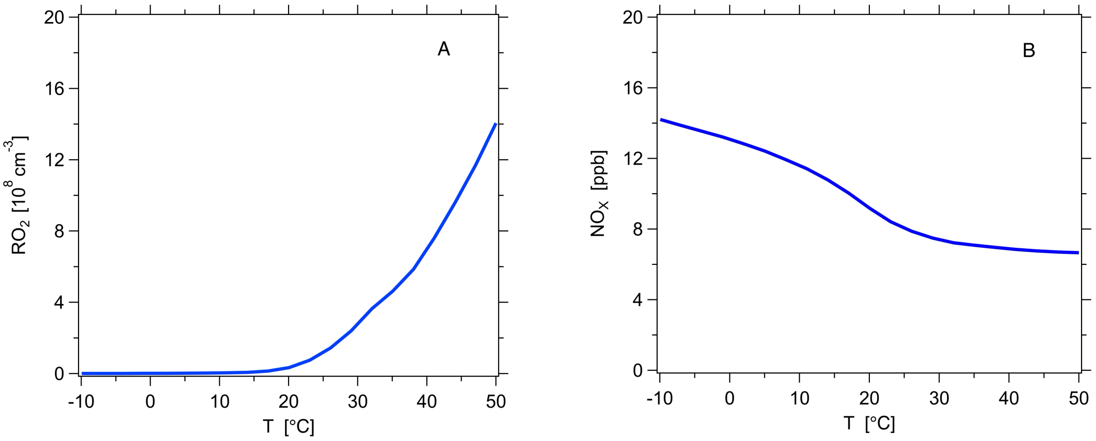

Figure S5. The box model-simulated RO_2 and NO_X concentrations at different temperatures.

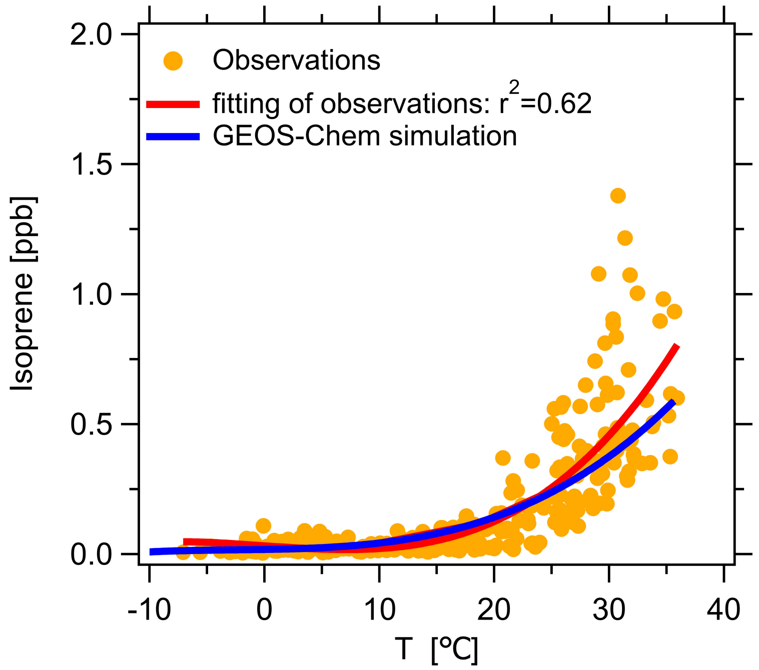

Figure S6. The dependence of isoprene on temperature in 2019, Beijing. Each dot corresponds to an observed daily daytime average. The red line is the fitting line of observations and blue line is the simulated result by the GEOS-Chem model.

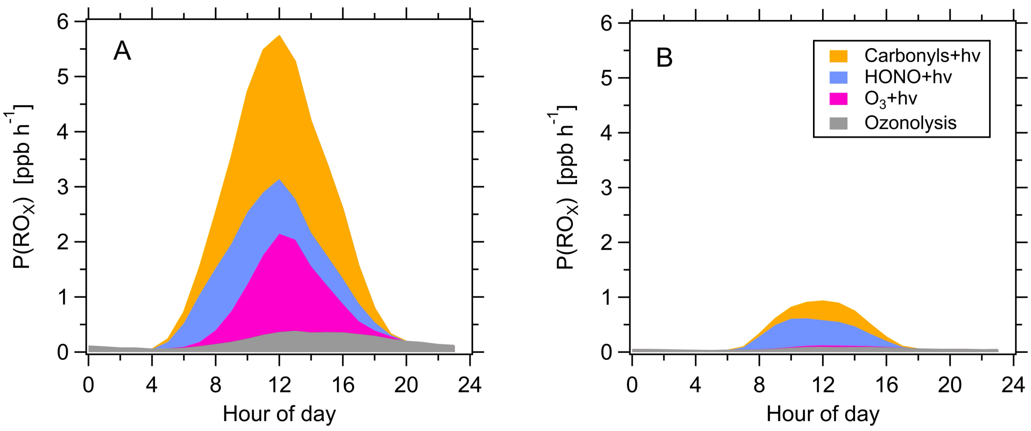

Figure S7. The average diurnal variation of the primary production of RO_X radicals ($P(RO_X)$) in summer (A) and winter (B) in Beijing. The results are simulated by the box model.

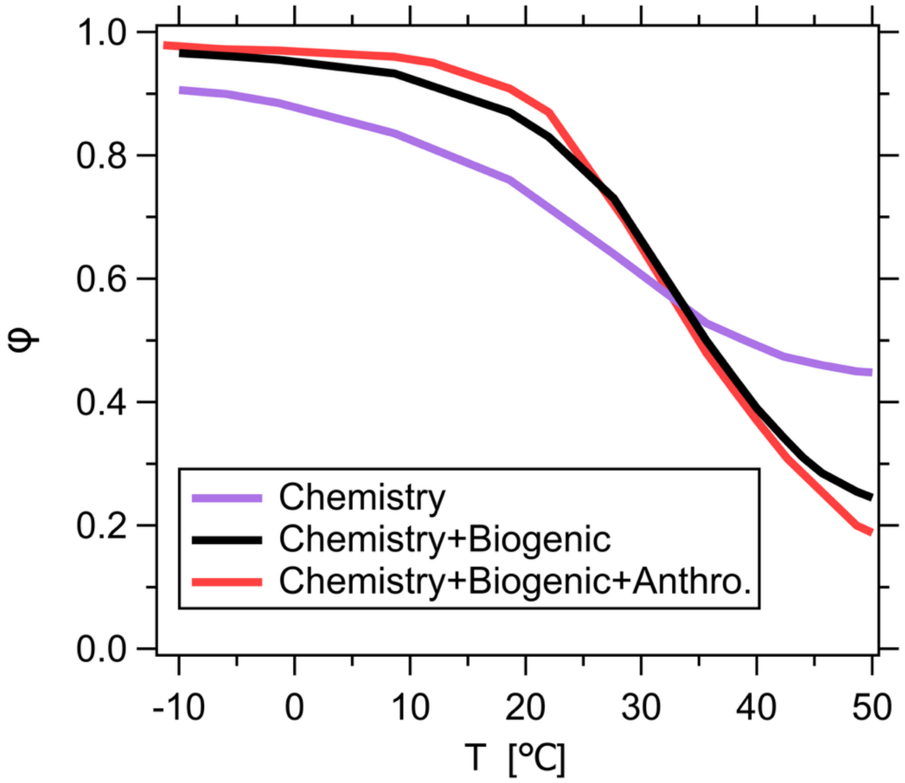

Figure S8. The temperature dependence of φ induced by chemistry alone (purple line), chemistry + biogenic emissions (black line), and chemistry + biogenic emissions + anthropogenic emissions (red line) in Beijing, which is simulated by the GEOS-Chem model.

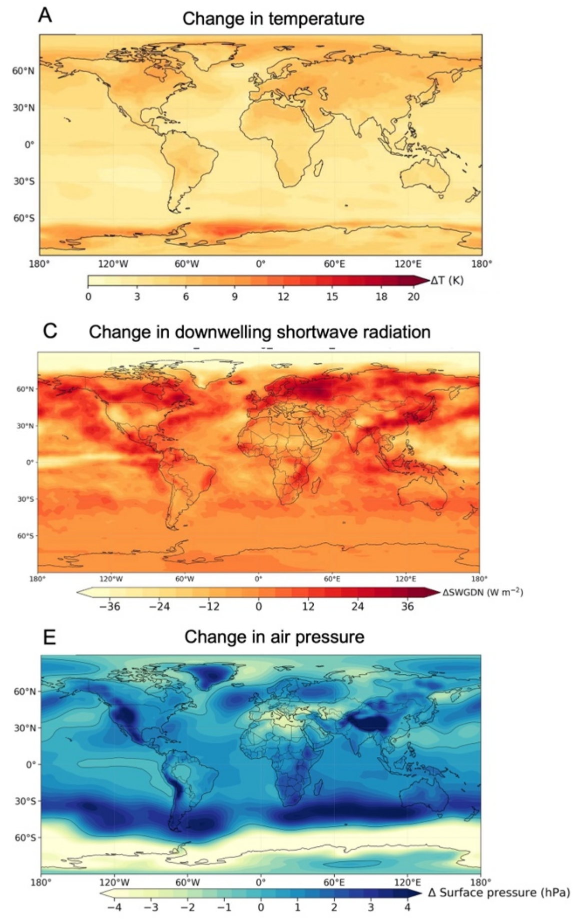

Figure S9. Changes in July meteorological parameters from 2019 to 2100. (**A**) mean surface temperature, (**B**) specific humidity, (**C**) downward shortwave radiation, (**D**) wind fields, and (**E**) surface air pressure, derived from CMIP6 simulations under the SSP5-8.5 scenario.

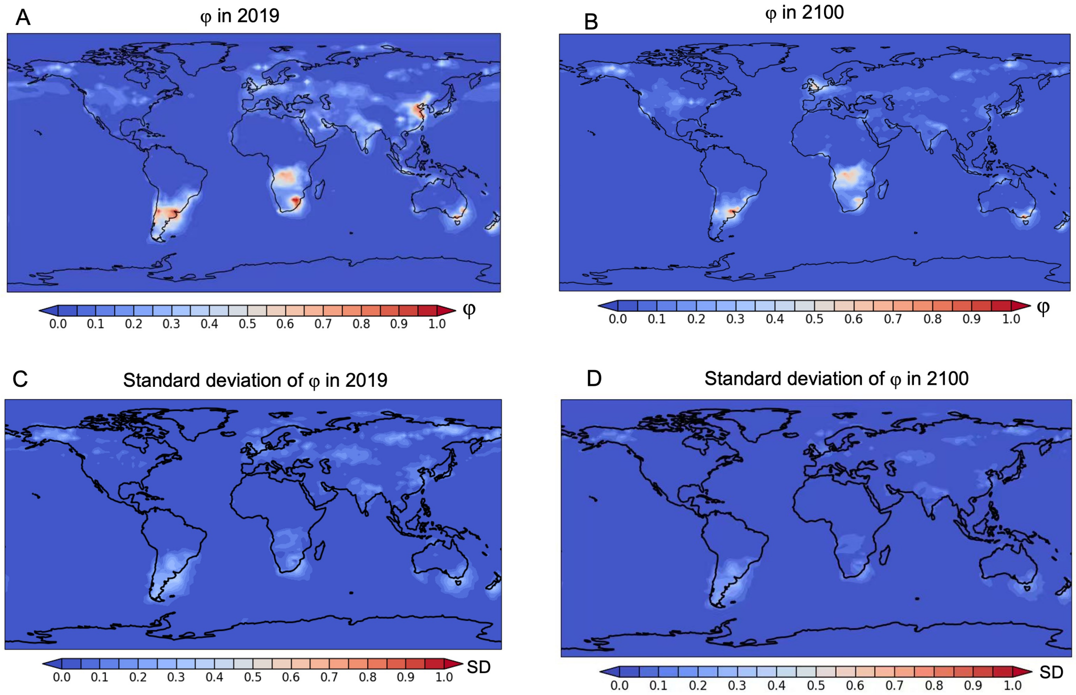

Figure S10. GEOS-Chem–simulated monthly daytime mean φ in July in 2019 (A) and in 2100 with changes in anthropogenic emissions and meteorology applied (B). Standard deviation of φ is shown in panels C and D.

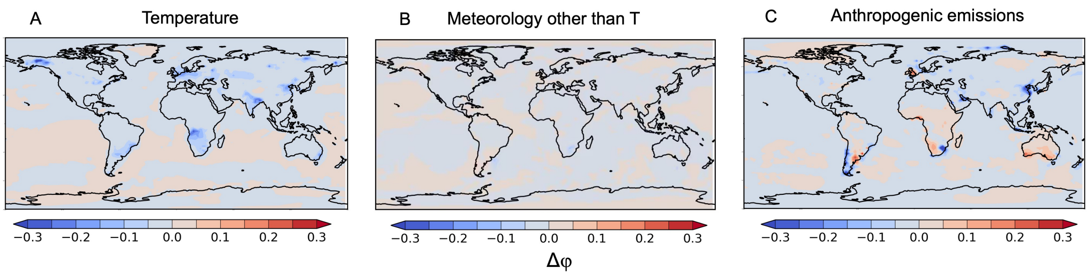

Figure S11. Individual contributions of changes in temperature (A), meteorological factors other than temperature (B), anthropogenic emissions (C) to the change in φ from 2019 to 2100 (Δφ). The results were simulated by GEOS-Chem model. Base on the conditions in July 2019, we simulate the future scenario for July 2100 by: (1) changing temperature alone to the condition in July 2100; (2) changing meteorology other than temperature to the condition in July 2100; (3) changing anthropogenic emissions to the condition in July 2100. The change in φ from 2019 to 2100 (Δφ) for the three scenarios were calculated, as shown in panels A, B and C respectively.

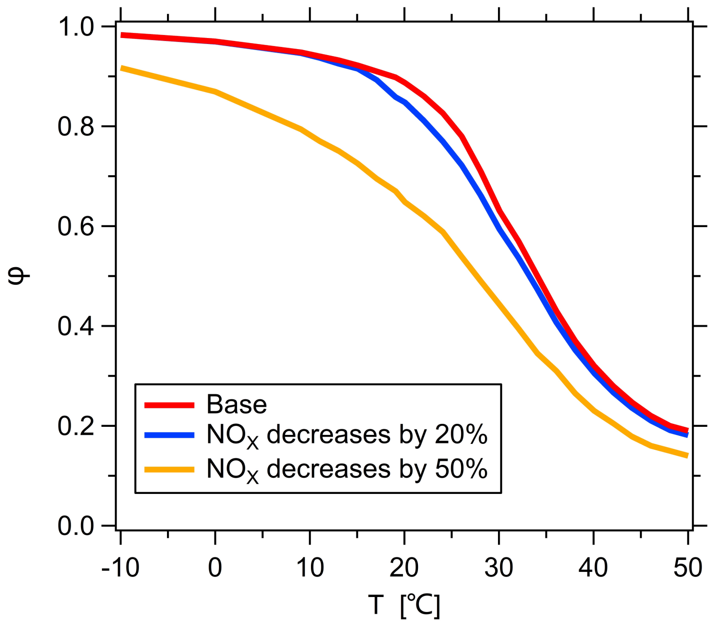

Figure S12. The temperature dependence of φ under the base scenario and the scenario with a decrease in NO_X by 20% and 50% in Beijing. The base scenario represents the conditions in summer 2019 in Beijing.

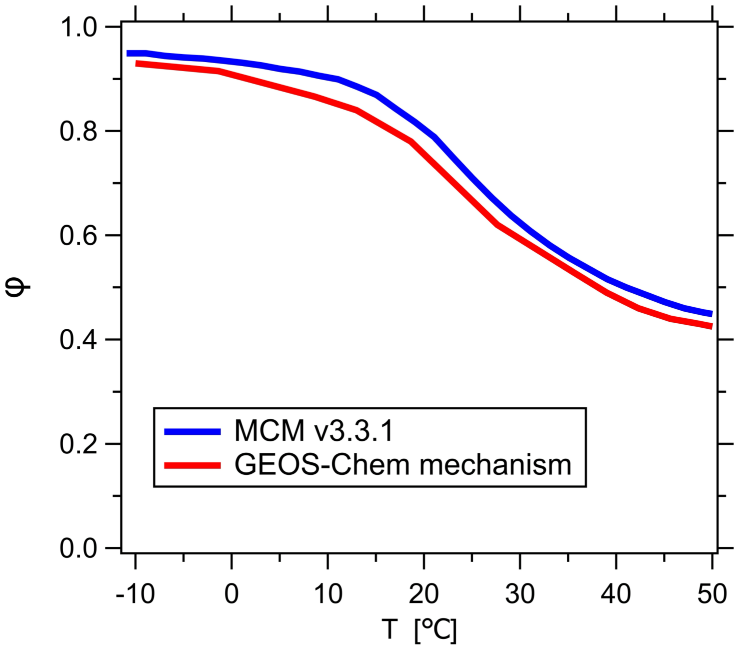

Figure S13. Temperature dependence of φ simulated by the box model using MCM v3.3.1 and the GEOS-Chem chemical mechanism, respectively.

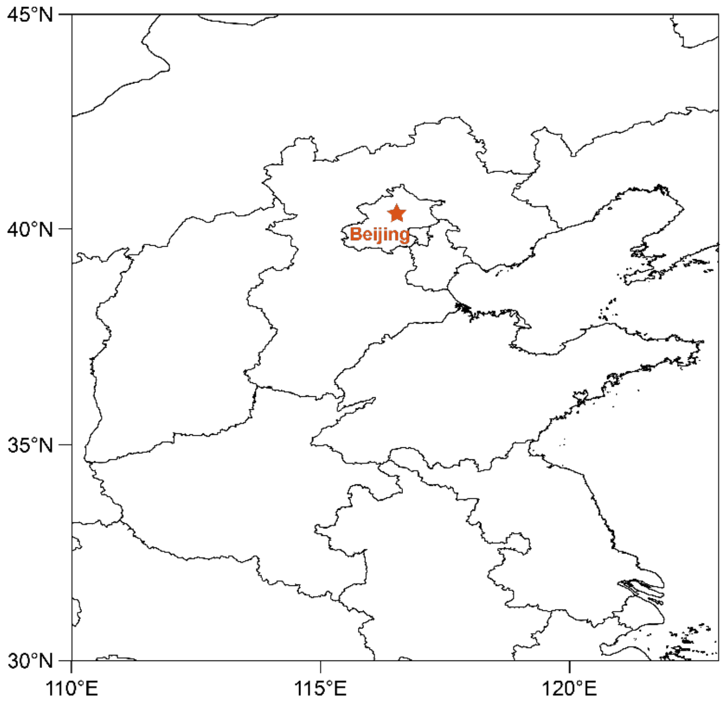

Figure S14. Domain over the North China Plain for the simulations with the FlexGrid capability of GEOS-Chem.

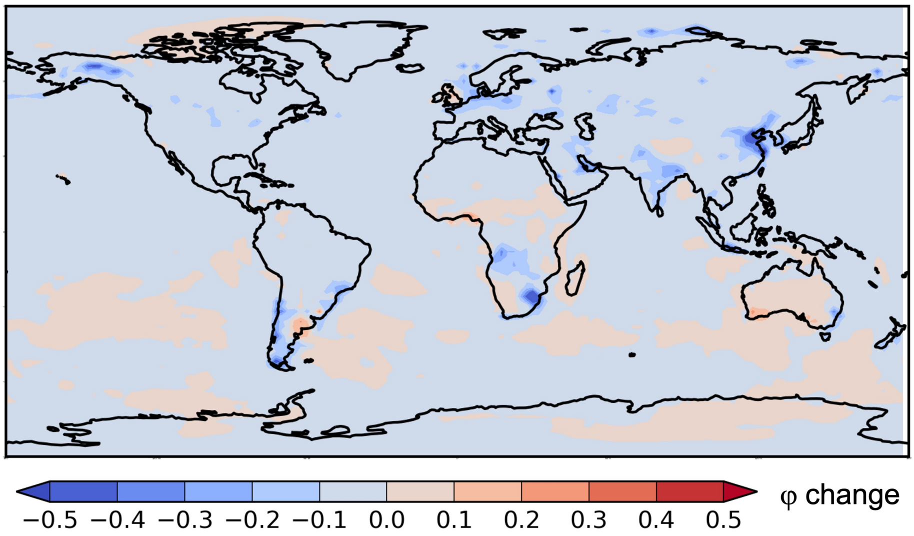

145 **Figure S15. Global distribution of the change in φ from 2019 to 2100 due to the**
146 **projected changes in meteorology and emissions.** The results are simulated by the
147 GEOS-Chem model.

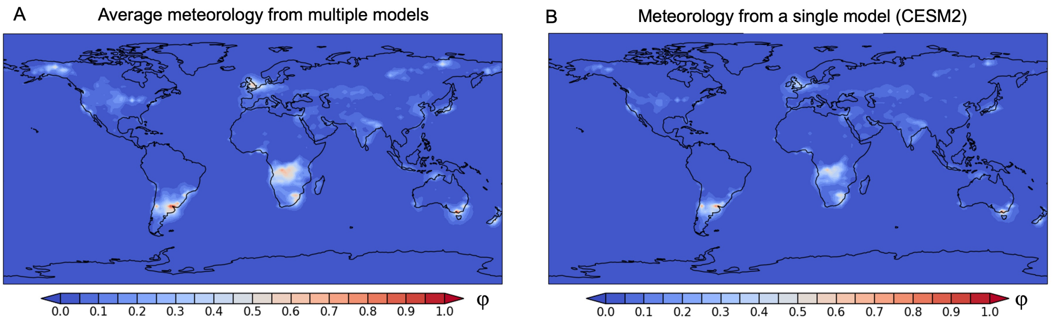

Figure S16. GEOS-Chem–simulated φ values for July 2100. The 2100 meteorology is generated by combining 2019 MERRA-2 data with CMIP6-derived meteorological increments from the average of multiple CMIP6 models (A) and from a single model (CESM2) (B).

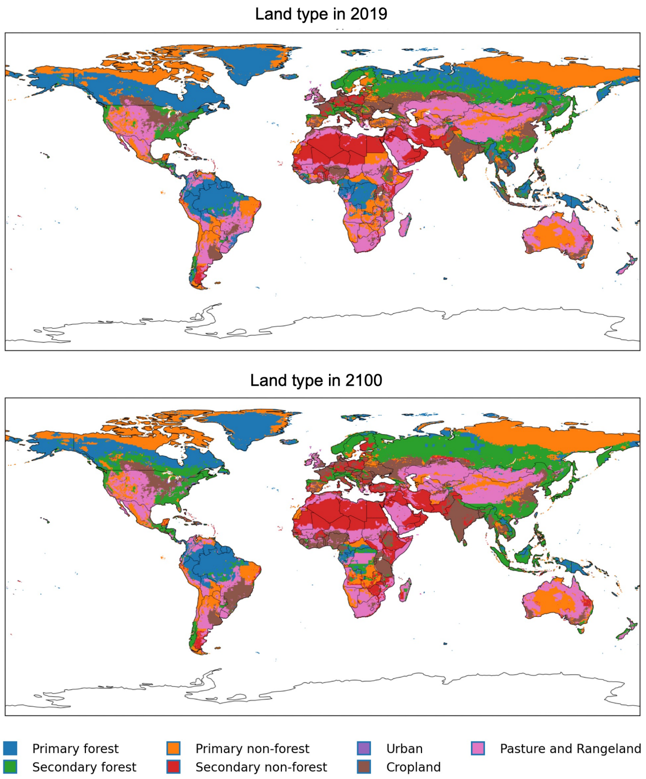

Figure S17. The land use type in 2019 and 2100 across the world. The data is derived from LUH2 v2f future harmonized land-use forcing datasets for CMIP6 (https://luh.umd.edu/data.shtml).

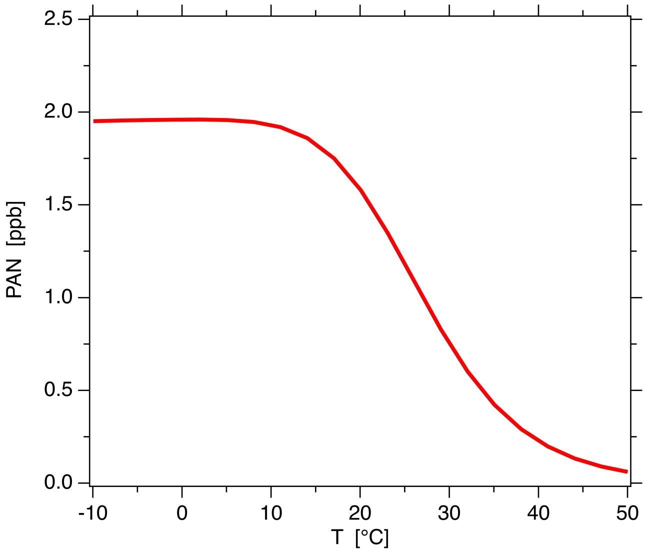

Figure S18. The box model-simulated PAN concentrations at different temperatures when the temperature dependence of the reaction rate constants of PANs is considered.

Table S1. Instruments deployed in the measurements undertaken in Beijing, Shanghai, and Wangdu between 2019 and 2020.

Parameters	Measurement technique	Time resolution	Detection limit	Accuracy
Photolysis frequencies	Spectroradiometer	10 s	/	± 10%
O_3	ultraviolet photometry	1 min	1 ppbv	± 5%
NO	Chemiluminescence	1 min	50 ppt	± 20%
NO_2	Chemiluminescence	1 min	50 ppt	± 20%
CO	infrared photometry	1 min	0.04 ppmv	± 5%
SO_2	Pulsed fluorescence	1 min	0.1 ppbv	± 5%
HCHO	Hantzsch fluorimetry	1 min	25 pptv	± 5%
C2-C10 VOCs	GC-FID/MS	1 h	20-300 pptv	± 15~20%
Oxygenated VOCs	PTR-MS	10 s	0.2 ppbv	± 15%
PANs	PANs-1000	5 min	50 pptv	/
$PM_{2.5}$	micro-oscillating balance	1 min	1 μg m^{-3}	± 5%
S_a	SMPS	1 min	/	±3%

* AOD: aerosol optical depth; SSA: single scattering albedo; AE: Ångström exponent.

Table S2. The measured species and measurement period of field campaign used in this study.

Measurement site	Period	Measured species	Measured radicals
Beijing	01.07.2019-31.07.2019	O_3, NO, NO_2, VOCs, HCHO, CO, SO_2, $PM_{2.5}$, aerosol optical properties, S_a, photolysis frequencies, meteorological factors	No measurement of radicals
Beijing	11.01.2016-28.02.2016	Same as above	OH, HO_2 and RO_2
Shanghai	01.07.2019-31.07.2019	Same as above	No measurement of radicals
Wangdu	07.06.2019-08.07.2019	Same as above	OH, HO_2 and RO_2

Table S3. Reaction rate constants of key reactions involved in termination and cycling of RO_X radicals, at summertime temperature (301 K) and wintertime temperature (276 K).

Types	Reactions	k at T=301 K (cm^3 $molec^{-1}$ s^{-1})	k at T=276 K (cm^3 $molec^{-1}$ s^{-1})	Relative change (%)
RO_X termination	$OH+NO_2 \rightarrow HNO_3$	9.6×10^{-12}	11.7×10^{-12}	22
	$OH+NO \rightarrow HONO$	9.5×10^{-12}	11.3×10^{-12}	19
	$RO_2+NO_2 \rightarrow PANs$	8.8×10^{-12}	10.0×10^{-12}	14
	$PANs \rightarrow RO_2+NO_2$	6.9×10^{-4}	1.04×10^{-5}	-98
	$CH_3O_2+NO_2 \rightarrow CH_3O_2NO_2$	5.7×10^{-12}	7.1×10^{-12}	25
	$CH_3O_2NO_2 \rightarrow CH_3O_2+NO_2$	2.1	0.10	-95
	$HO_2+NO_2 \rightarrow HO_2NO_2$	7.3×10^{-13}	8.7×10^{-13}	19
	$HO_2NO_2 \rightarrow HO_2+NO_2$	0.089	3.5×10^{-3}	-96
	$HO_2+HO_2 \rightarrow H_2O_2$	3.4×10^{-12}	6.0×10^{-12}	76
	$RO_2+HO_2 \rightarrow ROOH$ (e.g., $C_2H_5O_2+HO_2 \rightarrow C_2H_5OOH$)	7.7×10^{-12}	10.1×10^{-12}	31
RO_X cycling	$OH+VOCs \rightarrow RO_2$ (e.g., OH+isoprene$\rightarrow C_2H_5OOH$)	9.9×10^{-11}	11.1×10^{-11}	12
	$RO_2+NO \rightarrow HO_2+NO_2$ (e.g., $C_2H_5O_2+NO \rightarrow C_2H_5O+NO_2$)	8.9×10^{-12}	10.0×10^{-12}	12
	$HO_2+NO \rightarrow OH+NO_2$	8.5×10^{-12}	9.2×10^{-12}	8.2

Table S4. The comparison of observed and GEOS-Chem model-simulated MDA8 O_3 concentrations, NO_X concentrations and OH reactivity of VOCs in 2019 in Beijing. Mean bias (MB) and normalized mean bias (NMB) are given.

Species	Month	Observations (ppb)	Simulations (ppb)	MB (ppb)	NMB (%)
O_3	July	81	90	9.0	11
	February	37	33	-4.0	11
NO_X	July	18	19	1	5.6
	February	29	32	3.0	10
VOC^R	July	4.2	4.8	0.6	14
	February	3.5	3.9	0.4	11

Table S5. Estimated uncertainties in key reaction rate constants relevant to O_3 sensitivity, based on JPL evaluations (*26*).

Reactions	Uncertainty
$OH+NO_2 \rightarrow HNO_3$	15%
$OH+NO \rightarrow HONO$	20%
$RO_2+NO_2 \rightarrow PANs$	10%
$PANs \rightarrow RO_2+NO_2$	20%
$CH_3O_2+NO_2 \rightarrow CH_3O_2NO_2$	50%
$CH_3O_2NO_2 \rightarrow CH_3O_2+NO_2$	30%
$HO_2+NO_2 \rightarrow HO_2NO_2$	7%
$HO_2NO_2 \rightarrow HO_2+NO_2$	7%
$HO_2+HO_2 \rightarrow H_2O_2$	15%
$RO_2+HO_2 \rightarrow ROOH$	50%
$OH+VOCs \rightarrow RO_2$	10%
$RO_2+NO \rightarrow HO_2+NO_2$	15%
$HO_2+NO \rightarrow OH+NO_2$	10%

Table S6. Summary of input parameters used in the box model simulation. Hourly observations spanning entire days in February and July 2019 in Beijing were utilized in this study.

	February			July		
	Mean	Median	SD	Mean	Median	SD
CO [ppb]	693.43	664.92	412.35	434.82	404.04	199.43
NO_X [ppb]	27.10	20.00	21.83	18.11	14.29	11.80
NO [ppb]	6.87	1.68	14.17	1.03	0.29	2.16
NO_2 [ppb]	25.23	20.03	18.56	17.07	13.81	10.74
O_3 [ppb]	26.54	27.88	13.52	63.01	53.64	35.90
SO_2 [ppb]	2.75	2.21	2.07	0.21	0.14	0.23
TEMP [°C]	2.85	2.48	5.28	28.79	28.43	4.85
RH [%]	28.85	23.70	16.40	56.43	56.48	16.26
BP [mbar]	1017.94	1017.37	6.58	993.70	992.85	3.49
$j(O^1D)$ [s^{-1}]	2.09×10^{-6}	1.04×10^{-6}	2.09×10^{-6}	6.04×10^{-6}	1.32×10^{-6}	7.82×10^{-6}
$j(NO_2)$ [s^{-1}]	1.27×10^{-3}	4.92×10^{-6}	1.94×10^{-3}	2.56×10^{-3}	6.60×10^{-4}	3.16×10^{-3}
j(HONO) [s^{-1}]	2.09×10^{-4}	9.32×10^{-7}	3.20×10^{-4}	4.35×10^{-4}	1.10×10^{-4}	5.41×10^{-4}
$j(NO_3\_M)$ [s^{-1}]	4.33×10^{-3}	1.29×10^{-6}	6.34×10^{-3}	6.20×10^{-3}	1.62×10^{-3}	7.20×10^{-3}
$j(NO_3\_R)$ [s^{-1}]	3.39×10^{-2}	1.36×10^{-5}	4.99×10^{-2}	4.44×10^{-2}	1.39×10^{-2}	4.99×10^{-2}
j(HCHO_M) [s^{-1}]	4.27×10^{-6}	7.84×10^{-8}	6.65×10^{-6}	9.88×10^{-6}	2.31×10^{-6}	1.26×10^{-5}
j(HCHO_R) [s^{-1}]	2.99×10^{-6}	1.24×10^{-7}	4.72×10^{-6}	7.71×10^{-6}	1.63×10^{-6}	1.01×10^{-5}
$j(H_2O_2)$ [s^{-1}]	7.85×10^{-7}	3.01×10^{-8}	1.21×10^{-6}	1.89×10^{-6}	4.24×10^{-7}	2.42×10^{-6}
NMHCs [ppb]:						
ethane	5.3639	4.8085	2.6957	2.7617	2.6124	1.1439
ethene	2.8603	2.4695	2.2470	1.1980	1.0301	0.8739
propane	3.2394	2.6462	2.1359	2.8184	2.5872	1.5237
propene	0.4044	0.2901	0.3767	0.2086	0.1819	0.1502
iso-Butane	0.5889	0.5001	0.3984	0.7481	0.6786	0.4233
n-Butane	0.9854	0.8409	0.6606	1.2569	1.1417	0.7193
acetylene	2.4166	2.0280	1.7785	1.4786	1.3600	0.7944
trans-2-Butene	0.0156	0.0087	0.0195	0.0314	0.0311	0.0203
1-Butene	0.0625	0.0476	0.0590	0.0482	0.0453	0.0287
cis-2-butene	0.0321	0.0280	0.0314	0.0394	0.0397	0.0130
iso-pentane	0.5171	0.4374	0.4001	0.8713	0.7746	0.4986
n-pentane	0.2960	0.2526	0.2226	0.4302	0.3683	0.2810
1-3-Butadiene	0.0569	0.0332	0.0796	0.0139	0.0111	0.0133
1-Pentene	0.0094	0.0063	0.0088	0.0104	0.0086	0.0070
trans-2-pentene	0.0060	0.0017	0.0104	0.0009	0.0007	0.0009
isoprene	0.0245	0.0200	0.0191	0.2886	0.1645	0.3331
cis-2-pentene	0.0030	0.0010	0.0041	0.0016	0.0012	0.0015
2-2-dimethylbutane	0.0215	0.0200	0.0082	0.0219	0.0206	0.0092

2-3-dimethylbutane	0.0238	0.0180	0.0166	0.0294	0.0257	0.0151
2-methylpentane	0.1237	0.1017	0.0996	0.1922	0.1770	0.0975
3-methylpentane	0.1104	0.0911	0.0905	0.1996	0.1810	0.1025
1-hexene	0.0152	0.0100	0.0137	0.0109	0.0090	0.0071
n-hexane	0.1655	0.1260	0.1487	0.2820	0.2419	0.1807
2-methylhexane	0.0150	0.0130	0.0092	0.0205	0.0194	0.0077
cyclohexane	0.0523	0.0390	0.0494	0.0731	0.0610	0.0524
3-methylhexane	0.0340	0.0280	0.0242	0.0449	0.0400	0.0256
benzene	0.6507	0.6152	0.4678	0.4460	0.4010	0.2286
n-heptane	0.0511	0.0410	0.0397	0.0596	0.0529	0.0384
toluene	0.4654	0.3550	0.3924	0.5822	0.5039	0.3217
n-octane	0.0364	0.0300	0.0443	0.0393	0.0352	0.0206
ethylbenzene	0.0912	0.0660	0.0767	0.1385	0.1193	0.0806
nonane	0.0253	0.0209	0.0204	0.0280	0.0236	0.0152
m-p-xylene	0.2327	0.1610	0.2102	0.3691	0.3108	0.2366
o-xylene	0.0887	0.0598	0.0787	0.1461	0.1224	0.0906
styrene	0.0241	0.0140	0.0250	0.0197	0.0163	0.0133
isopropylbenzene	0.0100	0.0080	0.0067	0.0102	0.0093	0.0043
n-propylbenzene	0.0154	0.0132	0.0096	0.0197	0.0187	0.0073
m-ethyltoluene	0.0322	0.0255	0.0239	0.0445	0.0413	0.0221
p-ethyltoluene	0.0197	0.0164	0.0132	0.0256	0.0241	0.0111
n-decane	0.0235	0.0190	0.0188	0.0318	0.0289	0.0156
1-3-5-trimethylbenzene	0.0164	0.0132	0.0110	0.0205	0.0192	0.0093
o-ethyltoluene	0.0180	0.0150	0.0119	0.0230	0.0216	0.0101
1-2-4-trimethylbenzene	0.0478	0.0390	0.0368	0.0647	0.0590	0.0337
1-2-3-trimethylbenzene	0.0182	0.0159	0.0114	0.0222	0.0209	0.0092
n-undecane	0.0193	0.0158	0.0197	0.0456	0.0357	0.0370
n-dodecane	0.5216	0.5020	0.2614	0.0575	0.0544	0.0304

Table S7. The model input fields for GEOS-Chem simulations under different scenarios.

Scenarios	Meteorology	Anthropogenic emissions	Biogenic emissions
2019	Hourly data of MERRA-2 in 2019	CEDS emission inventory in 2019	Online MEGAN emissions, with vegetation distribution and meteorology in 2019.
2100:			
Scenario 1	The change in daily mean CMIP6 **meteorology** (temperature, humidity, solar radiation, air pressure, boundary layer height, wind fields) from 2019 to 2100 was added onto hourly data of MERRA-2 in 2019	CEDS emission inventory in 2019	Online MEGAN emissions, with vegetation distribution in 2019 and meteorology in 2100.
Scenario 2	The change in daily mean CMIP6 **temperature** from 2019 to 2100 was added onto hourly data of MERRA-2 in 2019	CEDS emission inventory in 2019	Online MEGAN emissions, with vegetation distribution in 2019 and temperature in 2100.
Scenario 3	The change in daily mean CMIP6 **non-temperature meteorology** (humidity, solar radiation, air pressure, boundary layer height, wind fields) from 2019 to 2100 was added onto hourly data of MERRA-2 in 2019	CEDS emission inventory in 2019	Online MEGAN emissions, with vegetation distribution in 2019 and non-temperature meteorology in 2100.
Scenario 4	Hourly data of MERRA-2 in 2019	CMIP6 emission inventory in 2100	Online MEGAN emissions, with vegetation distribution and meteorology in 2019.
Scenario 5	The change in daily mean CMIP6 meteorology from 2019 to 2100 was added onto	CMIP6 emission inventory in 2100	Online MEGAN emissions, with vegetation

	hourly data of MERRA-2 in 2019		distribution and meteorology in 2100.
Scenario 6	The change in daily mean **CESM2 meteorology** from 2019 to 2100 was added onto hourly data of MERRA-2 in 2019	CMIP6 emission inventory in 2100	Online MEGAN emissions, with vegetation distribution and meteorology in 2100.
